# Refined Procedure to Purify and Sequence Circulating Cell-Free DNA in Prostate Cancer

**DOI:** 10.3390/ijms26125839

**Published:** 2025-06-18

**Authors:** Samira Rahimirad, Seta Derderian, Lucie Hamel, Eleonora Scarlata, Ginette McKercher, Fadi Brimo, Raghu Rajan, Alexis Rompre-Brodeur, Wassim Kassouf, Rafael Sanchez-Salas, Armen Aprikian, Simone Chevalier

**Affiliations:** 1 Urologic Oncology Research Group, Cancer Research Program, Research Institute of the McGill University Health Center (RI-MUHC), Montreal, QC H4A 3J1, Canada; samira.rahimirad@mail.mcgill.ca (S.R.); seta.derderian@mail.mcgill.ca (S.D.); lucie.hamel@affiliate.mcgill.ca (L.H.); eleonora.scarlata@affiliate.mcgill.ca (E.S.); ginette.mckercher@affiliate.mcgill.ca (G.M.); alexis.rompre-brodeur@mcgill.ca (A.R.-B.); wassim.kassouf@mcgill.ca (W.K.); armen.aprikian@mcgill.ca (A.A.); 2 Department of Pathology, McGill University, Montreal, QC H3A 0G4, Canada; fadi.brimo@mcgill.ca; 3 Department of Oncology, McGill University, Montreal, QC H3A 0G4, Canada; raghu.rajan@mcgill.ca; 4 Department of Surgery (Urology Division), McGill University, Montreal, QC H3A 0G4, Canada; rafael.sanchez-salas@mcgill.ca; 5 Department of Medicine, McGill University, Montreal, QC H3A 0G4, Canada

**Keywords:** prostate cancer, liquid biopsies, cell-free DNA, circulating tumor DNA, whole genome sequencing

## Abstract

Cell-free DNA (cfDNA), a fragmented DNA circulating in blood, is a promising biomarker for cancer diagnosis and monitoring. Standardization of cfDNA isolation to enhance the sensitivity of molecular analyses in prostate cancer (PCa) is required. Towards this goal, we optimized existing methods to obtain a high quantity and quality of cfDNA from low volumes of plasma. The protocol was applied to samples from healthy males and three patient categories: radical prostatectomy (RP), disease-free (>6 years post-RP), and metastatic castration-resistant PCa (mCRPC). The yield was significantly higher in mCRPC cases, and the size of fragments was shorter. We compared for the first time library preparation using two cfDNA inputs and low vs. high sequencing depth. Clonal events were observed irrespective of input and depth, but lower input showed more subclonal events. The clinical application of the refined protocols to cfDNA samples from an mCRPC patient showed no tumor fraction before RP, while it increased to 25% at the advanced stage. Among chromosomal changes and mutations, the androgen receptor gene amplification was detected. Altogether, this comprehensive study on improved cfDNA procedures is highly promising to enhance the quality of liquid biopsy-based research for discoveries and much-needed clinical applications.

## 1. Introduction

Prostate cancer (PCa) is the second most common cancer in men throughout the world and is also among the leading causes of cancer death. Various risk factors are associated with PCa, including age, race, family history, lifestyle, diet, and physical activity [1]. PCa generally remains asymptomatic until it reaches an advanced stage. Therefore, early detection is crucial. Prostate tissue biopsy is currently the gold standard for diagnosing cancer and detecting histopathological anomalies, allowing the assessment of determinant parameters for clinical decision-making [2,3]. The blood levels of prostate-specific antigen (PSA) are also commonly used for the detection of PCa [4]. Radical prostatectomy (RP) and radiation therapy (RT) are effective curative treatments and are associated with a decrease in blood PSA to undetectable or nadir levels. However, rising PSA after curative therapies is an indication of biochemical recurrence (BCR) [4,5]. Other approaches for monitoring the disease include imaging modalities, which are non-invasive ways to reveal metastases in bone and soft tissues, but only when the cancer has progressed enough for detection [3,6,7]. Obtaining additional biopsies of prostate tumors from patients who did not undergo an RP and/or biopsies of metastases is not part of current practice for disease monitoring. Moreover, biopsies of metastases are invasive and have limitations, including low sensitivity and accuracy [8]. In contrast, obtaining liquid biopsies, such as blood, is minimally invasive. Blood contains various analytes from tumors, including tumor-educated platelets, circulating tumor (ct)DNAs/RNAs in cell-free (cf)DNAs/RNAs, extracellular vesicles, circulating tumor cells, microRNAs, and proteins. Genomic content can be released into the bloodstream from both healthy and tumor cells through apoptosis, necrosis, or active secretion. Therefore, isolated ctDNAs reflect genetic changes of tumors/metastases once released in liquid biopsies [9,10,11]. Liquid biopsies have the potential to complement diagnosis and are promising alternatives that could offer better understanding of tumor dynamics over the course of disease [10,12]. For instance, levels of cfDNA increase significantly in cancer patients, particularly at the metastatic stage [12]. Profiling ctDNA in liquid biopsies of PCa patients has been achieved using high-throughput approaches such as targeted and shallow whole-genome sequencing (WGS), showing mutations, copy number changes (CNVs), and gene fusions [13,14,15]. However, deep WGS of cfDNA from PCa patients is reported only in one study [16]. There are no guidelines on the best methods to obtain sufficient cfDNA for library preparation and sequencing. In this report, we summarize the methodology of cfDNA studies in PCa and present new findings improving existing purification protocols, library preparation, and WGS.

## 2. Results

### 2.1. Optimization of cfDNA Purification Improves cfDNA Quality and Yield Using Less Plasma

Studies reporting on cfDNA isolated from plasma of PCa patients include comparative genomic hybridization arrays, quantitative real-time PCR assays, targeted sequencing, and WGS (Table S1). Although almost all authors used Qiagen kits for isolation, there is no consensus on the volume of plasma to use as input. Also, high-depth sequencing studies (targeted or WGS) routinely used at least 6 mL of plasma to obtain enough cfDNA for library preparation and sequencing [14,16,17,18,19,20,21,22,23].

**Figure 1:**
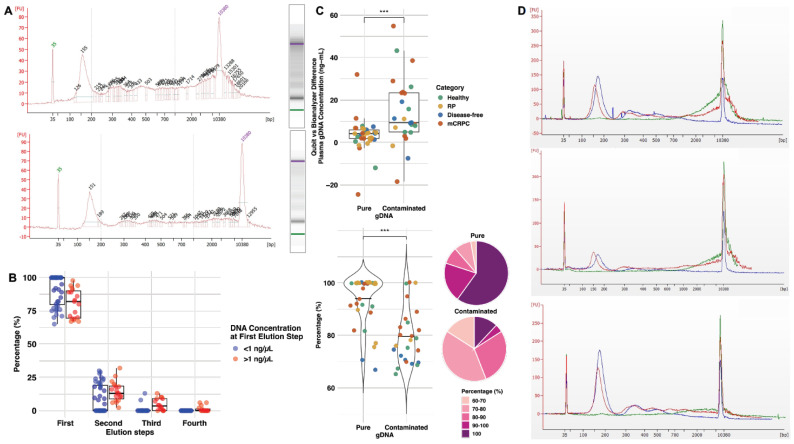
Purification protocol to obtain high quality cfDNA. (**A**). Bioanalyzer electropherograms and their corresponding gel electrophoresis of thawed plasma (3.6 mL) from an advanced patient, without (top panel) and with (lower panel) centrifugation. (**B**). Quantity of cfDNA collected (%) by elution step for low (blue, <1 ng/μL) vs. high (red, >1 ng/μL) DNA concentration (Qubit). (**C**). Differences between Qubit and Bioanalyzer readings were expressed in percentage and used to estimate the gDNA contamination in cfDNA samples from patients and healthy males. (Top) Bar graphs show the difference in the gDNA concentrations between samples considered “pure” vs. “contaminated” based on Bioanalyzer. (Bottom left) Violin plots show the significant difference of fragments (%) collected at first elution in pure vs. contaminated groups from the same samples. (Bottom right) Pie charts show percentages of DNA recovery at the first elution step in both pure and gDNA-contaminated samples. Statistically significant differences between groups are shown by *** *p* < 0.001. (**D**). Electropherograms showing the effects of magnetic beads to remove contaminating gDNA in plasma samples from three patients. Red line-sample before magnetic beads, blue line-pure cfDNA after magnetic beads, and green line-gDNA contamination remaining after magnets. Abbreviation: Base pair: bp; Cell-free DNA: cfDNA; Genomic DNA: gDNA; Metastatic castration-resistant prostate cancer: mCRPC; Radical prostatectomy: RP.

Our initial modification was to add proteinaseK before thawing a low volume of plasma (~3.6 mL), followed by centrifugation. This considerably helped to reduce the contaminating genomic DNA (gDNA), as shown by the decrease in high molecular weight material at the position of the upper marker (10,380 bp) in the Bioanalyzer (Figure 1A). Moreover, the recovery of a maximum quantity of high-quality cfDNA was achieved by performing four elution steps with warm buffer (56 °C) and increasing volumes to maximize yields/percentages (%) of cfDNA eluted at the first step. The total quantity of cfDNA was defined as the sum of short fragments, between 50 and 550 bp, representing mono-, di-, and tri-nucleosomes, detected in the Bioanalyzer electropherogram for each cfDNA elution. The cfDNA concentration was expressed relative to the volume of plasma (ng/mL). Results on seven plasma samples eluted with varying volumes of AVE buffer from the Qiagen kit show different cfDNA concentrations, with recoveries of 77–100% at the first elution step (Table S2). The choice of elution buffer to elute cfDNAs varies across PCa studies, with some researchers choosing AVE [22,24], while others used water [16,19,20]. We compared the performance of water with the same volumes on these samples, given that AVE contains sodium azide (NaN_3_), a chemical reported to inhibit library preparation for DNA sequencing [25]. Water was as efficient as AVE and led to the collection of at least 80–100% of cfDNA at the first elution step (average 92.9% for both) (Table S2). As the recovery of cfDNA with 85 μL of water was ≥94% in the first elute for four tested samples, further experiments were carried out with 85 μL of water added to the column at the first elution step, followed by 40 μL for the second step and 20 μL for the third and fourth steps. The median percentage of cfDNA fragments collected at the first elution step with water was 82% for concentrated samples compared to 100% for samples below 1 ng/μL (Figure 1B). These findings demonstrate that repeating elution steps at least three to four times is necessary to perform a complete isolation of cfDNA and increase yields. 

### 2.2. The Recovery of High-Purity cfDNA Reaches over 90%

Once refined, the protocol was applied to plasma samples from different categories of patients: prior to RP (*n* = 17), patients with no recurrence of disease (disease-free) for at least 6 years post-RP (*n* = 10), and patients at the mCRPC stage (*n* = 19), as well as healthy males (*n* = 14). Based on the Bioanalyzer results on gDNA (Figure 1A) and the processing of these additional samples, we observed that some were contaminated with fragments longer than 5000 bp. On the other hand, several preparations mainly contained small 50–550 bp fragments after four elution steps, reflecting a high degree of purity. We defined a threshold of >50 pg/μL of fragments longer than 5000 bp to call samples contaminated by gDNA vs. pure cfDNA. Due to the Bioanalyzer’s limited ability to quantify larger fragments migrating at the upper marker position, we used values from Qubit and Bioanalyzer to quantify the gDNA concentration in plasma. The median difference between Qubit and Bioanalyzer is presented in box plots, which show 35 pure samples with very low gDNA levels at 4.3 ng/mL and 25 others with significantly higher contamination at 10.7 ng/mL (*p* < 0.0002, Figure 1C). We next expressed DNA recovery as a percentage for each elution step. For pure samples (based on Bioanalyzer), we observed that most fragments (80–100%) were recovered at the first elution, as shown in pie charts (Figure 1C). In contrast, there was a wide distribution in contaminated samples, with lower recovery in the first elution (60–80%). The pie charts also show that a small portion of samples in the contaminated group were completely recovered in the first elution (100%). They were all at a low concentration of DNA (<1 ng/μL). The converse was true for pure samples of low recovery (60–70%), whose DNA concentrations were higher than 1 ng/μL. Overall, we estimated that the probability of collecting the majority of pure cfDNA fragments at first elution was 94% (average over all 60 samples), while it was 79.6% for contaminated samples (*p* = 7.7 × 10^−6^, Figure 1C, Violin plots), in agreement with data in Table S2. Therefore, gDNA contamination reduces the likelihood of collecting all circulating pure cfDNA fragments at the first elution step. To further reduce this contamination, a highly performant two-step wash with magnetic beads was added, allowing a complete removal of gDNA (Figure 1D). This procedure was reproducible and did not lead to noticeable losses of cfDNA (Figure S1). It was routinely used to remove contaminating gDNA when detected by Bioanalyzer. Altogether, our improved purification protocol yielded high quality and quantity of cfDNA using less plasma than previously reported (3.6 mL frozen plasma; Table S1). We highly recommend repeating elution steps four times to capture all cfDNA fragments and using magnetic beads to efficiently remove gDNA contamination and obtain pure cfDNA.

### 2.3. The Plasma of mCRPC Patients Contains Elevated cfDNA Levels Compared to Other Categories of Patients and Healthy Males

**Figure 2:**
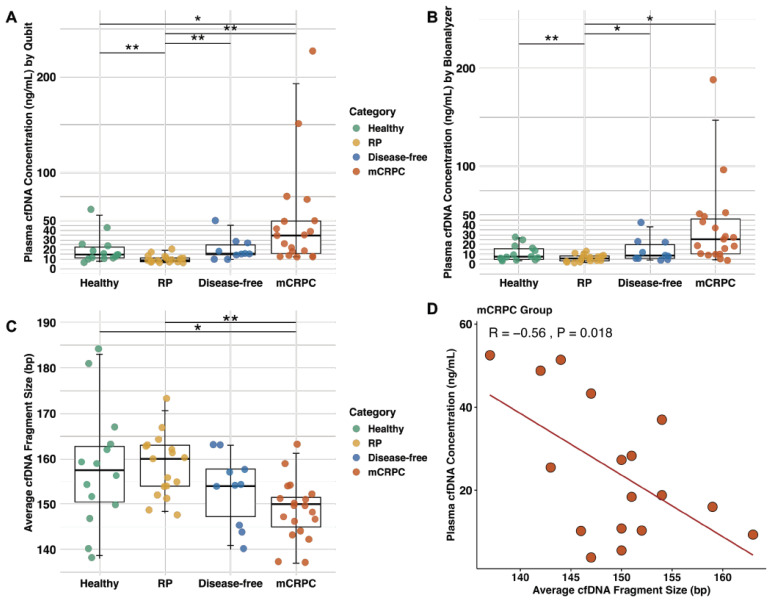
Comparison of cfDNA concentrations and fragment size in different categories of PCa patients and healthy males. Concentrations of cfDNA by categories of patients and controls are presented for (**A**) Qubit and (**B**) Bioanalyzer. (**C**). Average cfDNA fragment size (bp). Healthy: median = 158 bp (range = 138–184 bp); RP: median = 160 bp (range = 148–173 bp); Disease-free: median = 154 bp (range = 140–163 bp); mCRPC: median = 150 bp (range = 137–163 bp) (**C**). Statistically significant differences between groups are shown by * *p* < 0.05, ** *p* < 0.01. (**D**). Correlation between average cfDNA fragment size and concentration in mCRPC patients, with a Pearson coefficient (R) = −0.56. While two outliers were excluded in (**D**), their removal in (**A**,**B**) did not change the finding of significantly higher cfDNA concentrations in the mCRPC category. Abbreviations: Base pair: bp; Cell-free DNA: cfDNA; Metastatic Castration-Resistant Prostate Cancer: mCRPC; Radical Prostatectomy: RP.

With the optimized protocol, the plasma cfDNA concentrations of the above categories of patients and healthy men were measured by Qubit and Bioanalyzer. With both methodologies, the cfDNA concentration of mCRPC cases (median by Qubit = 34.5 and Bioanalyzer = 25.5 ng/mL) was the highest compared to other categories (Figure 2A,B). RP cases had the lowest quantity (median by Qubit = 8.6 and Bioanalyzer = 5.8 ng/mL). The overall plasma cfDNA concentration was comparable for healthy (median by Qubit = 14.6 and Bioanalyzer = 7.6 ng/mL) and disease-free cases (median by Qubit = 15.6 and Bioanalyzer = 8.3 ng/mL). This is supporting the Qubit overestimation of cfDNA quantity due to gDNA contamination in some samples (Figure 1C). Overall, the median plasma cfDNA concentration was lower in the healthy men (2.9 times, *p* = 0.02) and disease-free cases (2.6 times, not significant) than in mCRPC cases (22.2 ng/mL–without outliers).

### 2.4. The cfDNA Fragments from mCRPC Patients Are Smaller than Other Patient Categories and Healthy Males

The Bioanalyzer provides a global representation of cfDNA as mono-, di-, and tri-nucleosomes. The average cfDNA (mono-nucleosome) fragment size estimated for the first peak on the Bioanalyzer was ~50–250 bp. Healthy cases had a wider distribution of fragment sizes. Among PCa cases, the RP group had the largest fragments, while mCRPC had the lowest (*p* = 0.0016) (Figure 2C). A significant negative correlation was seen in the fragment size vs. plasma cfDNA concentration for the mCRPC group (Figure 2D). No association was found for the other groups (Figure S2). These findings imply that shorter cfDNA fragments are found in plasma samples containing more cfDNA.

### 2.5. Exploring cfDNA Concentration of Patients with Their Clinical Features

For all categories of cases and healthy males, there was no correlation of cfDNA concentration with age at time of blood draw (Figure S3). For patients, cfDNA concentration did not correlate with blood PSA prior to RP, but a positive correlation was found with blood PSA at inclusion for mCRPC patients (Figure S4). For all patients, we found no difference with tumor grade or stage (Figure S5). For patients from whom plasma was isolated before RP, no difference in cfDNA levels was observed with other pathologic features (Figure S6). For the 19 mCRPC cases, there was a trend for higher median cfDNA concentrations for the 12 cases under progression at the time of inclusion (excluding two outliers; Figure S7). There was no relationship with a history of having previous progression on Abiraterone and Docetaxel in mCRPC cases (Figure S8). Finally, overall survival was not significantly associated with high cfDNA levels (above the median at 22.2 ng/mL) (Figure S9). Further validation with more cases is required.

### 2.6. Further Protocol Refinements Lead to Highly Reliable Sequencing Data

**Figure 3:**
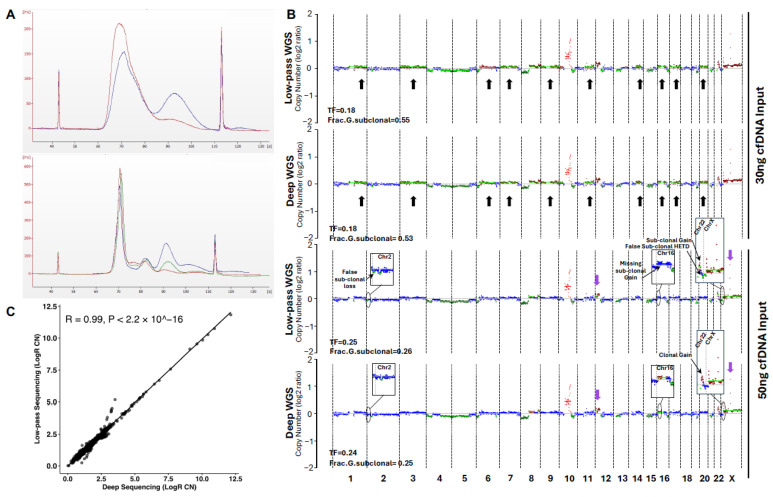
Optimizing conditions to prepare libraries and sequence cfDNA. cfDNA was isolated from 3.5 mL of plasma from a high-risk patient. (**A**). The cfDNAs of the Reference Standard and patient were amplified at different numbers of PCR cycles for library preparation, varying from 8 to 10 and 13. Electropherograms from the Bioanalyzer comparing, in the Top panel, the Reference Standard at 13 (blue) and 8 (red) PCR cycles; and in the Bottom panel, the patient sample at 13 (blue), 10 (green), and 8 (red) PCR cycles. (**B**). Chromosomal maps comparing the two cfDNA inputs at low-pass vs. deep WGS sequencing. The 1st and 2nd panels show CNVs (log2 ratio) for the 30 ng input at low pass (1st panel; post-alignment depth of 9.7×) and deep (2nd panel; 147×). The 3rd and 4th panels show CNVs for the 50 ng input at low-pass (3rd panel) and deep (4th panel) WGS. Black arrows indicate subclonal events. Blue dots in rectangle boxes drawn for Chr2, Chr16, Chr22, and ChrX represent neutral CN; light and dark red show amplification and clonal gain; dark green stands for loss, and light green lines are subclonal events. Purple arrows show clonal events at 30 ng input detected as subclonal at higher input. (**C**). Correlation between LogR CN for low-pass and deep WGS for the 50 ng input sample, yielding a Pearson coefficient (R) of 0.99 and *p* < 2.2 × 10^−16^ (^ represents the power). Abbreviations: Cell-free DNA: cfDNA; Copy number variations: CNVs; The fraction of the genome that harbors subclonal alterations: Frac.G.subclonal; Heterozygote deletion: HETD; Polymerase chain reaction: PCR; TF: Tumor fraction; WGS: Whole Genome Sequencing.

Recovering elevated levels of high-quality cfDNAs from small volumes of plasma is key for patients with aggressive disease donating blood for research and for downstream molecular applications. The quantity of cfDNA for library preparation and the sequencing depth have not been thoroughly investigated and are critical before initiating translational projects on plasma from PCa patients throughout the course of their disease. Pilot experiments were conducted to optimize the number of PCR cycles to prepare sequencing libraries. Skipping fragmentation in the TruSeq Nano DNA kit protocol was among the steps considered in collaboration with Illumina specialists. No further changes were applied at this point. Amplification was tested at 8 and 13 PCR cycles for the cfDNA Reference Standard (30 ng) and at 8, 10, and 13 cycles for the PCa cfDNA sample. Eight PCR cycles had the lowest off-target amplification peak (~85–105 s, equal to 500–2000 bp), as seen in both PCa samples (Figure 3A, bottom) and the Reference Standard (Figure 3A, top), compared to 10 and 13 cycles with false amplifications of larger fragments. This had no effect on library concentrations as determined by qPCR, with similar values at different numbers of cycles (Table S3). The number of PCR cycles selected for additional samples was 8 to avoid non-specific amplification of large fragments.

Most studies report on ~30 ng of cfDNA to use for library preparation. For the first time, two sequencing libraries were prepared to compare 30 and 50 ng as inputs for the same cfDNA sample from an mCRPC patient. In parallel, each of these cfDNA inputs was subjected to both low-pass (10× depth) and deep (170×) WGS for higher sensitivity. At low depth, the sample with 30 ng cfDNA input had a noticeable number of false subclonal events compared to 50 ng input (Figure 3B-black arrows). Other subclonal events (Chr12, ChrX) were found as clonal events with the 50 ng input (Figure 3B-purple arrows). As expected, the 30 ng input had a lower tumor fraction, 0.18 (1st and 2nd maps), compared to 0.24–0.25 with the 50 ng input (3rd and 4th maps). At high depth, the 30 ng input sample showed results similar to low pass (1st and 2nd maps). CNVs detected with the 50 ng input at low pass were also comparable at high depth (Figure 3B-3rd and 4th maps). This infers that higher cfDNA input improves sequencing data. A more in-depth comparison of a 50 ng input sample at low-pass and deep sequencing revealed high correlations and similarities in clonal events (Figure 3C). Nonetheless, discordances were still detected for subclonal events. At low-pass WGS, some segments were detected as subclonal CNVs, while at higher depth they were neutral (Figure 3B, Chr2, ChrX). Furthermore, a clonal event at high depth was detected as a subclonal event at low pass (Figure 3B, Chr22). Also, a subclonal CNV detected at high depth was not captured at low depth (Figure 3B, Chr16).

**Figure 4:**
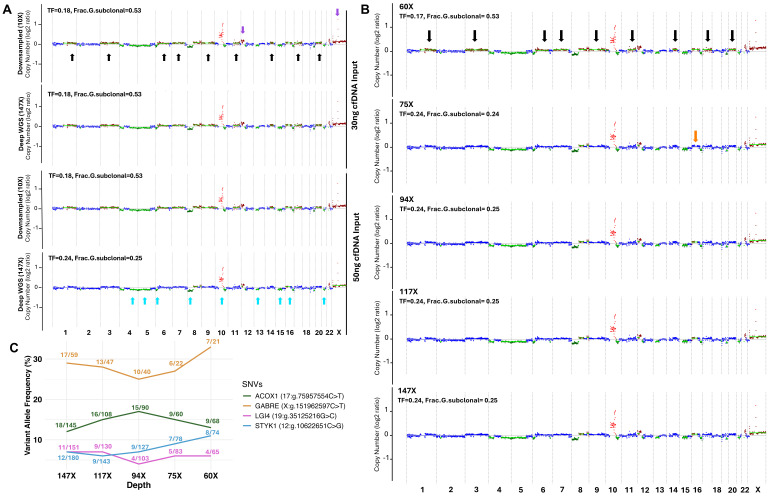
Gradual downsampling defines the optimum depth of sequencing. Attempt to reproduce findings at low pass and identify the minimum sequencing depth (**A**). Chromosomal maps showing CNVs after downsampling the 30 ng and 50 ng inputs deep WGS bam files (~147×) to reach 10× coverage. Turquoise arrows show events detected in all inputs and coverage. Black arrows show subclonal events not detected in deep WGS with 50 ng. Purple arrows show clonal gains, which were detected as subclonal events at high input and depth. (**B**). Chromosomal maps comparing CNVs detected at different depths of sequencing, including the original bam file with 50 ng input at 147× depth and gradual downsampling to 117×, 94×, 75×, and 60×. Black arrows show subclonal gains detected at 60× while absent at greater depth. The orange arrow represents a missing subclonal gain at chromosome 16 when downsampled at 75×. (**C**). Percentages of variant allele frequency of SNVs at deep sequencing (147×) and gradual downsampling (~117×, 94×, 75×, 60×). The number of reads harboring the mutations out of the total reads is indicated for each gene at different depths. Abbreviations: Cell-free DNA: cfDNA; Copy number variations: CNVs; The fraction of the genome that harbors subclonal alterations: Frac.G.subclonal; Single nucleotide variations: SNVs; Tumor Fraction: TF.

To reproduce the comparison of low-pass and high-depth sequencing, the deep sequencing data was downsampled three times to reach about 10× in depth for both cfDNA inputs (Figure 4A, only showing 10× downsampled data for one of the files for each input; other files can be seen in Figure S10). Regardless of inputs, all downsampled bam files showed the same pattern of alterations. Clonal CNVs at 10×, including chr10 amplification and chr8 loss, were similar to high-depth data. This was also true for the subclonal losses at chromosomes 4, 5, 6, 10, 12, 13, 15, 16, and 21 (Figure 4A-turquoise arrows). A great number of false subclonal gains were detected in all downsampled data (chromosomes: 1, 3, 6, 7, 9, 10, 11, 14, 17, and 20) (Figure 4A-black arrows), which were absent in deep sequencing with 50 ng input. While gains in chrX and part of chr12 were detected as subclonal at high depth, they were annotated as clonal when downsampled at 10×, which is the equivalent to low depth (Figure 4A-purple arrows). This further supports that high depth of sequencing can better reflect the true clonality of alterations. Of note, downsampling the 50 ng to 10× reduced the tumor fraction from 0.24 to 0.18, while the fraction of subclonal events increased in parallel (Figure 4A).

To track genomic alterations detected in deep WGS and identify the optimal depth of sequencing, we gradually downsampled the 50 ng input (20% reduction each time). The original depth of sequencing for the sample after alignment and post-alignment was 147×. Thus, the bam file was downsampled to reach 117×, 94×, 75×, and 60× (Figure 4B). All clonal and subclonal events were detected at 117× and 94×. There was one subclonal event missing on Chr16, which was not detected at 75× (Figure 4B-orange arrow), while it was found at 60×. False subclonal events were detected at 60× and the CNV pattern was similar to downsampled 10× bam files (Figure 4B-black arrows). These findings confirm the superiority of higher input and sequencing depth.

For SNVs, the literature reports that Mutect2 and Vardict are the most common tools used to call mutations in cfDNAs. In most cfDNA targeted sequencing studies, criteria considered to call mutations are: at least 10 supporting reads, variant allele frequency (VAF) above 1%, mutant allele fraction (MAF) 20 times higher than the background error rate (i.e., the average allele fraction across all germline DNA samples) and 3 times higher than the allele fraction in the paired germline DNA [14,18,19,20,26,27,28,29]. Some criteria differ in cfDNA WGS; for instance, at least 8 supporting reads, MAF ≥ 10%, MAF 50 times higher than the background error rate, and 10 times higher than in the matched germline sample [16]. In our study, we performed variant calling using four tools, including Mutect2, Vardict, Varscan, and Strelka2. Mutations were included if: (I) they were detected by at least two out of four tools; (II) they had a minimum depth of 10×; (III) they had a minimum VAF of 5%; (IV) they had at least 8 supporting reads in cfDNA and 0 reads in matched germline blood DNA; and (V) they had a population allele frequency below 0.01% in 1000 genomes, gnomad, kaviar, and ExAc. All variants detected as ‘benign’ or ‘likely benign’ were excluded. Of interest, SNVs were identified in the 50 ng input. They were detected in *ACOX1* (17:g.75957554C>T), *GABRE* (X:g.151962597C>T), *LGI4* (19:g.35125216G>C), and *STYK1* (12:g.10622651C>G) both in the original 147× and downsampled bam files at different depths ~117×, 94×, 75×, and 60× (Figure 4C). However, there were some fluctuations in the VAF of mutations when gradually downsampling bam files. Our findings indicate that more than 8 supporting reads require a coverage of at least 117× to detect low VAF mutations in *LGI4* (19:g.35125216G>C) and *STYK1* (12:g.10622651C>G) (Figure 4C). SNVs cannot be detected at low pass, irrespective of input quantity. Collectively, these CNV and SNV results indicate that it is preferable to use the highest quantity possible of cfDNA to prepare libraries. Furthermore, high-depth sequencing is the best option when precious samples are sequenced to ensure true clonality of alterations and to identify significant mutations.

### 2.7. Clinical Application of the Overall Refined Protocol

**Figure 5:**
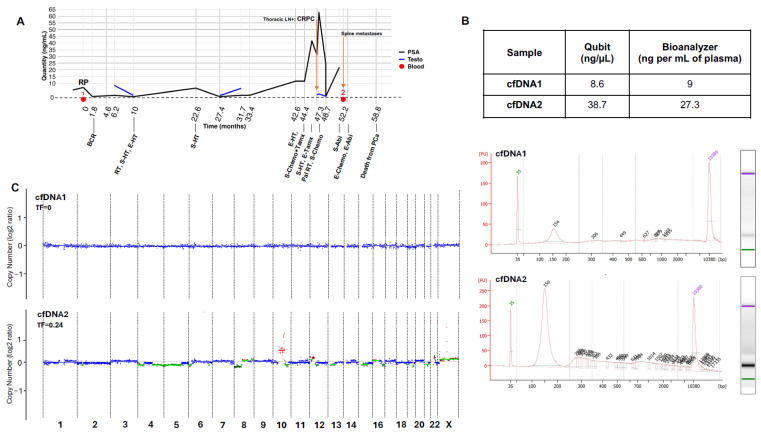
Refined protocol to isolate and sequence cfDNAs from plasma obtained at diagnosis and at the advanced stage of PCa. (**A**). Trajectory of high-risk PCa patient from prior to RP until death. The black and blue lines show blood levels (ng/mL) of PSA and Testosterone, respectively. Red dots indicate times of blood collections used to purify plasma cfDNA1 (prior to RP) and cfDNA2 (6 months prior to death). (**B**). Determination of cfDNA1 and cfDNA2 quantity and quality. Top panel: cfDNA concentrations assessed with Qubit and Bioanalyzer. Middle and Bottom panels: Electropherograms and corresponding gel electrophoresis from Bioanalyzer of cfDNA1 and cfDNA2, respectively. (**C**). WGS of cfDNA1 and cfDNA2 at 170×. TF is indicated on the top left. Blue dots represent neutral CN, light and dark red show amplification and clonal gain; dark green stands for loss, and light green lines are subclonal events. Abbreviations: Abiraterone: Abi; Biochemical recurrence: BCR; Cell-free DNA: cfDNA; Chemotherapy: Chemo; End: E; Palliative: Pal; Prostate Specific Antigen: PSA; Radical prostatectomy: RP; Radiotherapy: RT; Tamoxifen: Tamx; Start: S; Testosterone: Testo; Tumor Fraction: TF; Whole Genome Sequencing: WGS.

Given the above findings, we applied the optimized protocol to two plasma samples collected from a high-risk PCa patient. His features at diagnosis were GG 5 (Gleason score 9 (4 + 5)), pT3a staging, negative margins, and no lymph node extension. His overall trajectory was drawn based on his PSA levels over the course of the disease (Figure 5A). Briefly, he had a rapid recurrence, received standard of care based on progression, and died within 5 years.

The first blood draw (cfDNA1) was obtained prior to RP, and the second, cfDNA2, 6 months prior to death. Sample cfDNA2 had 3–4 times higher concentration than cfDNA1 (Figure 5B, top). Results by Qubit and Bioanalyzer suggest minimal gDNA contamination, as confirmed by the electropherogram (Figure 5B, bottom).

The yield of cfDNA1 from 3.6 mL of plasma was low at 30 ng compared to 130 ng for cfDNA2. To increase the quantity of cfDNA1 to prepare the sequencing library, the remaining 1.75 mL of plasma was extracted. Finally, 40 ng of cfDNA1 and 50 ng of cfDNA2 were used to generate libraries for deep WGS.

At the time of blood collection prior to surgery, no CNVs were detected in cfDNA1, which meant no tumor fraction (Figure 5C). In cfDNA2, clonal CNVs and subclonal events were detected with tumor fraction of ~0.24. As mentioned above, the main CNVs were copy number loss at chr8 as well as gains at chr10 and chr12, detected as clonal events. Noticeably, *AR* amplification was detected at chrX. The SNVs consist of four new mutations, as described above in *ACOX1* (17:g.75957554C>T), *GABRE* (X:g.151962597C>T), *LGI4* (19:g.35125216G>C), and *STYK1* (12:g.10622651C>G). Collectively, these findings show that despite being a high-risk RP patient with a short overall survival, there was no tumor fraction in his initial cfDNA. Significant changes occurred during progression to mCRPC, which resulted in a detectable tumor fraction with both CNVs and SNVs in the sample collected late in his trajectory.

## 3. Discussion

For the first time, in this present investigation, we reviewed in depth and modified the cfDNA isolation and sequencing protocols in the context of PCa to provide guidelines. The optimized methods were next applied to the plasma from several patients and to a high-risk patient.

Collection and analysis of cfDNA require specialized approaches due to the short half-life and low quantity of DNA fragments, particularly at the early stage of disease. Blood processing must be done carefully to keep blood cells intact, due to the possibility that germline DNA may contaminate cfDNA through normal blood cell lysis. We report first the modifications made to the QIAGEN cfDNA isolation kit, including adding proteinaseK before thawing the plasma, replacing the AVE elution buffer with water, and repeating the column elution step four times with different volumes of water to ensure the complete recovery of cfDNA. This implies that significant quantities of cfDNAs likely remain on the column when the elution is carried out with only a small volume of AVE buffer and one elution step. The quantity of cfDNA obtained with the optimized procedure, requiring generally 3.6 mL of plasma from most PCa patients and healthy males, is in line with cfDNA values obtained when isolating cfDNA from 7 mL up to 55 mL, but without considering gDNA contamination [30]. This would support higher recovery of cfDNA with our procedure with consistent plasma volume. Variability in plasma volume directly impacts the cfDNA yields. Normalizing the cfDNA concentration to the volume of plasma (ng/mL) is essential to reflect biological variations rather than differences arising from sample processing. Our results also revealed that there is a limited chance of complete isolation of cfDNA at the first elution step when samples contain quantities higher than 1 ng/μL. Genomic DNA contamination reduces the possibility of collecting all cfDNA at the first elution step. Magnetic beads were reported to reduce such contamination [31,32]. We not only accounted for gDNA in cfDNA samples by comparing Qubit and Bioanalyzer data but also by removing the contaminating material with magnetic beads to obtain “pure” cfDNA preparations. We provided a reliable threshold for considering samples as pure or contaminated when they contain >50 pg/μL of fragments longer than 5000 bp in the Bioanalyzer. Accordingly, we propose a method to quantify cfDNA by summing only peaks between 50 and 550 bp, representing mono-, di-, and tri-nucleosomes.

We next isolated cfDNA from banked plasma of 60 individuals, including healthy males and PCa patients at the time of RP, disease-free, and at the mCRPC stage. There was no correlation of cfDNA concentration with clinical features at diagnosis. While blood PSA levels were not correlated with cfDNA concentration in RP, disease-free, and mCRPC patients at the time of diagnosis, an association was observed between levels of cfDNA and PSA at the time of inclusion in the study for mCRPC cases. Our findings are consistent with previous reports with a larger sample size that compared the cfDNA quantity among healthy controls, RP cases, and mCRPC patients [22,30,33]. Authors showed higher plasma cfDNA in mCRPC patients compared to RP cases and/or healthy males. It is noticeable that the lowest cfDNA quantity we detected was in the RP cases whose blood was drawn in the operating room prior to anesthesia. It is thus unlikely that lower cfDNA levels may be due to anesthetic agents reported to reduce the cfDNA quantity by affecting cell survival/death [30,34]. Therefore, cfDNA levels may be low at the early stage of PCa and at the time of surgery [30]. It was reported that the cfDNA concentration in mCRPC patients is directly affected by treatment, the amount being significantly decreased (3.89%) in responders to Abiraterone acetate and prednisone but slightly increased (0.94%) in non-responders as compared with pre-treatment levels [35]. In our study, it was not possible to state differences in cfDNA quantity based on treatments including Abiraterone, Enzalutamide, and Docetaxel, as too few patients were included. A more systematic study on mCRPC patients is required. Of interest, elevated cfDNA concentration was related to progression-free survival [36].

Among features characterizing cfDNAs is the size of fragments detected by the Bioanalyzer. When analyzed among our patients, mCRPC samples had the shortest fragments, correlating with the highest plasma concentration of cfDNA. This finding is in support of levels of cfDNA increasing and fragment size being reduced during cancer progression [37]. Our study showed no difference between fragment size in the RP vs. healthy groups, whereas a lower average cfDNA fragment size was reported in RP patients compared to healthy males [30].

In the field of PCa, the majority of cfDNA studies use targeted sequencing or low-pass WGS. Various types of library preparation kits have been used, the most common being Roche NimbleGen SeqCap EZ Choice and ThruPLEX Plasma-seq. Targeted sequencing studies focused on less than 10 genes [28] and up to about 570 genes [24]. Only one study performed cfDNA deep WGS, but for samples showing ctDNA fraction greater than 30% in targeted sequencing of a panel of 73 PCa-relevant genes [16]. To our knowledge, the best conditions to prepare libraries with an adequate number of PCR cycles, cfDNA input, and depth of sequencing have not been systematically examined. After skipping the fragmentation step in the TruSeq DNA Nano library prep protocol to be compatible with the short size of cfDNA (recommendation by Illumina), we investigated how the quantities of cfDNA affect library preparation and results of both low-pass and deep WGS. We report for the first-time that the best number of PCR cycles is 8; with 10 or 13 PCR cycles, a false positive amplification of libraries occurred at 500–2000 bp. Of note, library preparation with 8 PCR cycles was efficient irrespective of samples being treated with a magnet. Using the cfDNA sample of an mCRPC case, we showed that with a higher input, there is a lower chance of detecting false subclonal CNVs. This is in line with libraries generated for targeted sequencing with low input (below 20 ng), where there are higher levels of PCR duplicates, but excluding them resulted in unique reads correlating positively with cfDNA concentration [38]. Sequencing artifacts such as PCR amplification errors can mimic true mutations, especially at low allele frequencies, which may lead to inappropriate clinical decisions. Our two libraries (30 and 50 ng inputs) were sequenced at low-pass (10×) and at high depth (170×). Although all clonal CNVs were detected in both inputs at low and deep sequencing, differences were found with regard to subclonal events. There were subclonal CNVs detected at high depth but not at low depth. Also, due to low coverage, false subclonal events were found at 10× depth. We thus downsampled the high-depth bam files from 147× (depth after alignment) to 117×, 94×, 75×, and 60×. False subclonal events started to be detectable at 60×, and SNV analysis revealed that at least 117× coverage is required to detect mutations with low allele frequency. These findings indicate that while deep sequencing is much more expensive than low depth, it provides more accurate CNV results as well as SNVs, which are not possible to identify at low depth. On the other hand, if the aim of a study is only clonal CNVs, low-pass WGS would be the most affordable choice.

As mentioned, collecting a sufficient quantity of cfDNA at an early stage of PCa and at the time of surgery can be challenging [30], as observed in our RP series. To move further with optimized cfDNA protocols and investigate the clinical significance of the two samples obtained for the high-risk patient, more plasma (5.3 mL) was required to isolate 40 ng of cfDNA prior to RP, whereas, as expected, 3.5 mL of his plasma yielded 130 ng of cfDNA at the late stage of disease. The estimation of tumor fraction in the plasma refers to the proportion of tumor DNA relative to total cfDNA. Library preparation and high-depth sequencing revealed no tumor fraction in the cfDNA of the first blood draw of our patient but several genomic alterations when progressing to the advanced mCRPC stage. Therefore, while ctDNA shows potential for aiding disease monitoring through analyzing genomic alterations, further studies with larger case numbers are required to validate this conclusion. The tumor fraction is mainly dependent on tumor type and disease burden and ranges from less than 1% to greater than 30% [26]. Consistent findings were reported on genomic alterations present in the ctDNA of PCa patients and DNA from metastatic biopsies, ranging between 90% and 94%, whereas none of the alterations found in ctDNA were detected in primary prostate tumors [14,29]. This is in line with the absence of ctDNA in the cfDNA1 (sample before RP), although he was a high-risk case. Of note, it was reported that up to 96.9% of somatic mutations identified in time-matched ctDNA were shared with metastatic tissue biopsies of mCRPC patients [16]. Consistent with this finding, authors reported that truncal alterations in metastatic biopsies are detected as subclonal in ctDNA and derived from multiple metastases. Furthermore, when analyzing genomic ctDNA alterations in relation to treatment, a higher frequency was observed from before starting Abiraterone or Enzalutamide until the end of treatment in non-responders compared to responders [39].

The most interesting genomic alteration we identified is *AR* amplification, which has been reported in several ctDNA studies. For instance, it was frequently found by targeted sequencing at a high depth (500×–10,000×) in cfDNA and biopsies of metastatic tissues [40,41,42] and of primary prostate tumors from advanced cases [43]. Such amplification was detected in ctDNA of 55.4% of patients collected at disease progression [43]. Moreover, it was related to treatment and identified by digital droplet PCR in 14.3% of patients a month before starting first-line treatment with androgen receptor signaling inhibitors (ARSIs), like Abiraterone or Enzalutamide [21], whereas 5.3% (1 out of the 19) of patients showed *AR* amplification in cfDNA when blood samples were collected during ARSI response [43]. It is noticeable that *AR* amplification is found at higher frequency in patients progressing on Enzalutamide compared to Abiraterone or other agents and is thus associated with resistance to Enzalutamide [18,40,44]. The copy number gain varies from <1 to more than 150 copies, depending on disease stage and treatment response or non-response [16,26,43]. Shorter time to progression was reported in patients with *AR* gain that have more than 8 copies compared to patients with less than 8 copies [26]. Along with this *AR* amplification, *AR* enhancer region amplification was detected in 40% of mCRPC patients. Both progression-free survival and overall survival are significantly shorter among patients with both *AR* gene and enhancer amplifications compared to those without [41]. The survival rate in patients with baseline *AR* amplification at 10-, 20-, and 30-months post-chemotherapy was 100%, 53%, and 0%, respectively, while in patients without baseline *AR* gain, the survival rate was 96%, 80%, and 58%, respectively [45]. Overall, *AR* amplification has the potential of detecting treatment resistance over the course of the disease.

Studies also indicate that ctDNAs can reflect the landscape of clonally expanded driver mutations, even when the fraction of metastatic source is minimal. High concordance was detected in *AR* amplification with other CNV calls in genes such as *BRCA2*, *ATM*, *PTEN*, *PIK3CA*, *TP53*, and *RB1* in the ctDNA and matched solid biopsy [14,18,46,47,48]. These were not found in our patient, although a subclonal *PTEN* loss was observed.

The *AR* is known to be highly mutated. One study reported that 83.3% (10/12) of cases showed shared mutations in genomic DNA from prostate and ctDNA of CRPC patients [43]. In the ctDNA of our high-risk case isolated at the late stage and sequenced at high depth, there was no mutation in the *AR* gene. However, four new mutations were detected in *STYK1*, *ACOX1*, *LGI4*, and *GABRE*. The *STYK1* gene was found to be overexpressed in CRPC patients [49]. This gene was reported to promote metastasis by activating MEK/ERK and PI3K/AKT signaling in human hepatocellular carcinoma and by reducing PINT2/HAI-2 expression in non-small cell lung cancer [50,51]. Higher expression levels of *ACOX1* were detected in PC3 cells, a cell line with prostatic small cell carcinoma, compared to 22RV1 cells (a human prostate carcinoma epithelial cell line) [52]. Direct studies on *LGI4* or *GABRE* in PCa are limited. *LGI4* is known as a putative secreted protein involved in neural development and function [53]. *GABRE* encodes a subunit of the gamma-aminobutyric acid (GABA)-A receptor, which is involved in inhibitory neurotransmission in the central nervous system. GABA has been shown to promote gastrin-releasing peptide secretion in neuroendocrine-like PCa cells, contributing to tumor progression [54]. Additionally, another subunit of the GABA-A receptor, *GABRB3*, has been identified as a potential biomarker in PCa [55]. Further research is necessary to determine whether these mutations have any significant role in PCa.

Overall, this study provides a comprehensive literature review on cfDNA methodology applicable to PCa, with step-by-step optimization of protocols for cfDNA purification, library preparation, and sequencing. Comparison of cfDNA quantity and fragment size among healthy controls and patients at the time of RP or at mCRPC is in line with previous reports. We also added one more group, disease-free patients, who had similar quantity and fragment size compared to healthy males. The highest levels and shorter cfDNA fragments were detected in mCRPC patients. The inclusion of cfDNA1 and cfDNA2 from the high-risk patient suggests the effectiveness of cfDNA levels and size in monitoring disease over time. The current work presents pros and cons with low-depth sequencing, which is more common in studying cfDNA. High depth definitively proved superiority for detecting true copy number variations and mutations if inputs are optimal. Taken together, the optimized cfDNA procedure will hopefully help in guiding the standardization of assays to better contribute to meaningful clinical applications, thereby opening the way to further experimentation on larger sample sizes.

## 4. Material and Methods

### 4.1. Established Protocols on cfDNA in PCa

A literature review was conducted to collect cfDNA studies in PCa and summarize the methods used. The screening was done between 2015 and 2025 through Pubmed, Web of Science, and Scopus databases. The following combinations of keywords were used: prostate cancer, cancer of the prostate, prostatic cancer, cancer of the prostate, prostate neoplasm, prostatic neoplasm; circulating tumor DNA, cell-free tumor DNA, cell-free DNA; liquid biopsy, plasma, serum, and blood. Abstracts and review studies were excluded, and duplicated articles were eliminated. 45 studies were kept. The complete steps in methods from isolation up to sequencing and bioinformatics analysis are presented in the Supplementary Table S1.

### 4.2. Ethical Review and Patient Consent 

This study was approved by the Ethics Review Board of the McGill University Health Center (MUHC) (MP-37-2017-3189/MUHC biobank; MP-37-2021-6957: multicentric Quebec PROCURE PCa Biobank), in conformity with the Declaration of Helsinki. Written consent was obtained from participants donating blood for research.

### 4.3. Blood Germline DNA Processing and Sequencing

Whole blood was collected in EDTA tubes and centrifuged at 2500× *g* for 15 min at room temperature within 2 h after collection. Plasma (1.8 mL aliquots) and buffy coat layers were stored at −80 °C until analysis. Germline DNA from buffy coats was extracted using a QIAamp DNA kit (Qiagen; Germantown, MD, USA), quantified by picogreen assay (Thermo Fisher Scientific; Waltham, MA, USA), and stored at −80 °C. The quality of DNA was confirmed by agarose gel electrophoresis. DNA samples with high quality and sufficient quantity were sent to the McGill Genome Center (MGC) for preparing a library with the Lucigen NxSeq AmpFREE kit (Biosearch Technology; Middlesex, UK). Quality was tested using Tapestation (Agilent; Millcreek, ON, Canada). Libraries were sequenced at a depth of 40× for blood DNA on the Novaseq6000-S4 using 150-bp paired-end sequencing reads (2 × 150 bp; Illumina).

### 4.4. Isolation and Quantification of cfDNA

The QIAamp circulating nucleic acid kit (Qiagen) was used for isolating cfDNA from plasma with some modifications applied to the protocol. In this study, cfDNA was isolated from 2 tubes of 1.8 mL plasma. 90 μL of proteinaseK was added to each tube before thawing at room temperature for 15 min. Each tube was gently vortexed three times during this period. Tubes were centrifuged at 12,000 rpm for 5 min at 4 °C. After centrifugation, 3.68 mL of plasma from the two tubes was transferred into a 50 mL tube to which 0.32 mL of PBS was added (total 4.0 mL). This was followed by the second addition of 220 μL proteinaseK and 3.2 mL ACL buffer (containing 1 μg carrier RNA) and vortexed gently for 30 s. The mixture was incubated for 30 min at 60 °C for a better recovery of DNA after protein digestion and lipid solubilization. The ACB buffer (7.2 mL) was added to the mixture, vortexed thoroughly for 30 s, and incubated on ice for 5 min. The lysate was passed through the QIAamp mini spin column during approximately 13 min under a pressure of −900 mbar, as applied by the QIAvac 24 Plus vacuum system. In line with the manufacturer protocol, ACW1 (600 μL) buffer followed by ACW2 buffer (750 μL) and 100% ethanol (750 μL) were passed through the column sequentially, next placed in a 2 mL collection tube, and centrifuged at 20,000× *g* for 3 min at room temperature. It was incubated at 56 °C for 10 min. Different volumes of warm water (56 °C) were used for elution instead of AVE buffer, as explained above when preparing the library. It was applied in the center of the column, incubated at room temperature for 3 min, and centrifuged at 20,000× *g* for 1 min. The elution step was repeated 4 times, and nucleic acids were recuperated into separate aliquots. Elution volumes for each step were 85 μL (elute #1), 40 μL (elute #2), 20 μL (elute #3), and 20 μL (elute #4). After gentle pipetting, 2 μL cfDNA aliquots were tested for quality and determining quantity. cfDNA aliquots were stored at −20 °C.

The DNA concentration was measured on a Qubit 3.0 Fluorometer (ThermoFisher Scientific) using a Qubit dsDNA HS assay Kit according to the manufacturer’s protocol. To evaluate the quality and precise quantity of cfDNA, 1 μL of sample from each aliquot was run on the Bioanalyzer2100 (Agilent) using the high-sensitivity DNA kit. Based on peaks in the Bioanalyzer electropherogram, cfDNA fragments were categorized into four groups according to their size, including 50 to 250 bp, 250–350 bp, 350–550 bp, 550–5000 bp, and >5000 bp for gDNA contamination. The total quantity of fragments within the 50–550 bp range was determined by summing the quantities across the four eluates, unless otherwise mentioned in the Result section. Their sum was used to report on cfDNA concentrations (ng) per mL of plasma.

### 4.5. Purification of cfDNA by Removal of Contaminating gDNA

When Bioanalyzer showed evidence of gDNA contamination, a size selection procedure was applied to remove large fragments using AMPure XP magnetic beads (Beckman; Coulter, Brea, CA, USA) in 96-well plates. The first step was carried out using 40 μL of sample and 20 μL of AMPure beads (0.5×), incubated at room temperature for 5 min, and maintained on the magnetic stand (ThermoFisher Scientific) for 3 min. The supernatant was collected in a separate well and mixed thoroughly with 120 μL of magnetic beads (2×). The incubation at room temperature and magnetic stand step was done a second time for 5 min. A 70% ethanol wash (with 200 μL) was performed twice, and the liquid was removed. The magnetic beads were dried in air for 5 min. Then, 40 μL of water was added to the beads and placed on a magnet to collect pure cfDNA in the elute. The gDNA was extracted from the beads and tested on the Bioanalyzer along with the cfDNA to validate the procedure.

### 4.6. cfDNA Library Preparation and Sequencing

Modifications were brought to the TruSeq Nano DNA low-throughput library prep kit (Illumina; San Diego, CA, USA) protocol, with help from Illumina, to render this kit compatible for cfDNA. The modified protocol was first tested on 30 ng of Multiplex I cfDNA Reference Standard Set (Horizon Discovery, Waterbeach, UK) as a positive control and 30 ng from one of our isolated cfDNA samples. The fragmentation step was skipped for both the cfDNA Reference Standard Set and our cfDNA sample. Repair-end was performed by adding 40 μL of ERP mix to 50 μL of cfDNA sample to convert the natural single-strand ends into blunt ends. Selection library size was done in only one step through adding 2× SPB magnetic beads provided in the kit and removing the supernatant after 6 min on the magnetic stand. Beads were washed two times with 70% ethanol, then diluted with 20 μL of RSB buffer. Adenylate 3′ ends and ligate adapters steps were performed according to the manufacturer’s instructions, and IDT for Illumina–TruSeq DNA UD Indexes v2 (Illumina) were used for index oligo ligation. To amplify cfDNA libraries, 8–10 and 13 PCR cycles were compared to find the optimal number of cycles. Library concentrations were tested by qPCR using the sparQ Universal Library Quant Kit (Quantabio; Beverly, MA, USA), as per the manufacturer’s protocol.

Library quantification was carried out through both our Bioanalyzer and the Tapestation at the MGC sequencing facility. Libraries were built from one sample with two cfDNA inputs. Pooled libraries were sequenced on Novaseq6000-S4 using 150-bp paired-end sequencing reads (2 × 150 bp; Illumina) at low depth (~10×) and high depth (~170×).

### 4.7. Sequence Data Analysis

The Genpipe pre-built pipelines were used in this study. Genpipe is a Python-based platform developed at the Canadian Center for Computational Genomics (C3G) that provides various genomic sequencing pipelines, including for WGS [56]. The tumorpair pipeline version 4.6.0 was adapted, and additional steps were added for analyzing cfDNA WGS. Details of data analysis will be further explained below.

For sequence alignment and quality control, adapters were trimmed using Skewer [57], and the end quality and mean quality values were set to 25. Paired-end reads were aligned against the hg38 reference genome using Burrows-Wheelers Aligner (BWA-MEM) version 0.7.17 [58] and realigned by GATK-realigner [59]. Mark duplication was done using Picard version 2.23.3. Recalibration was performed through GATK BaseRecalibrator. The Conpair version 0.2 was used to estimate contamination and sample concordance [60].

### 4.8. Analysis of CNVs

The CNV calling was performed using IchorCNA [61] for both cfDNA low-depth and deep sequencing. Sequencing data of DNA extracted from the blood buffy coat layer was used as a source of germline DNA.

### 4.9. Analysis of Somatic Mutations

SNVs were called using four variant callers, including Mutect2 [62], Strelka2 [63], Vardict [64], and Varscan2 [65]. An ensemble vcf file of variations from four tools was developed using Bcbio.variation.recall. Then, GATK VariantAnnotator was used for annotating variants. The five criteria we considered to call true somatic variations were mentioned in the Result section. To further exclude false-positive results, all mutations were manually reviewed using Integrative Genomics Viewer (IGV) [66].

### 4.10. Downsampling

The Sambamba version V0.8.2 [67] tool was used to downsample the aligned, de-duplicated, recalibrated bam file. Samtools version V1.20 [68] was used to estimate the average depth of sequencing.

### 4.11. Statistical Analysis

The cfDNA concentration quantified by Qubit or Bioanalyzer and fragment size were compared among healthy controls, patients at the time of RP, disease-free, and mCRPC patients using the Wilcoxon rank sum test. Also, the Wilcoxon test was used for comparison of cfDNA concentration by Gleason Grade (GG) of RP, disease-free and mCRPC cases, pathologic features at RP (intraductal carcinoma, lymphovascular, lymph node or seminal vesicle invasions, pathological staging, and surgical margins), and progression at inclusion or progression on Abiraterone, Enzalutamide, or Docetaxel treatments for mCRPC cases.

For comparing continuous characteristics, Pearson correlation coefficients were used, including cfDNA concentrations versus percentages of fragments collected at first elusion or versus age at diagnosis, PSA at biopsy or inclusion in the study, or average cfDNA fragment size (bp). The mCRPC cases were categorized into low or high cfDNA levels based on the median plasma cfDNA concentration. The association between cfDNA levels and overall survival of mCRPC patients was evaluated by Kaplan-Meier plot. The correlation between low-pass and high-depth sequencing data was assessed by Pearson correlation coefficients. All data analyses were performed using R version 4.4.2 [69].

## Data Availability

Data can be made available upon request.

## Supplementary Materials

The following supporting information can be downloaded at: https://www.mdpi.com/article/10.3390/ijms26125839/s1.
