# Supplementary material for: Refined Procedure to Purify and Sequence Circulating Cell-Free DNA in Prostate Cancer
Source: Int J Mol Sci. 2025 Jun 18;26(12):5839. doi: 10.3390/ijms26125839 (PMC12192578; doi:10.3390/ijms26125839)
Supplement: Supplementary file 1 [file ijms-26-05839-s001.zip › Legends of Supplementary Figures and Tables.pdf]

Legends of Supplementary Figures

Figure S1. cfDNA recovery after magnetic bead treatment. The Bioanalyzer electropherograms representing the contaminating cfDNA in 12 samples, with genomic DNA (red line) and pure cfDNA after magnetic treatment (blue line). No substantial cfDNA loss was noticed. Abbreviations: Fluorescent unit: FU; Second: S.

Figure S2. Investigating the relationship between cfDNA fragment size and plasma cfDNA concentrations in healthy males, RP patients, and disease-free cases. There was no correlation between these characteristics in all three groups. Abbreviations: Base Pair: bp; Cell-free DNA: cfDNA; Radical prostatectomy: RP.

Figure S3. Determination of whether the cfDNA concentrations in the plasma of healthy males and different categories of patients correlate with age. The cfDNA concentrations in the plasma of these individuals are not associated with age, irrespective of outliers, being analyzed (A) or removed (B). Abbreviations: Cell-free DNA: cfDNA; Metastatic castration-resistant prostate cancer: mCRPC; Radical prostatectomy: RP.

Figure S4. Determination of whether the cfDNA concentrations in the plasma of different categories of patients correlate with blood PSA levels. The cfDNA concentration is not associated with blood PSA pre-RP in RP, disease-free, and mCRPC patients, irrespective of outliers in the analysis (A) or their removal (B). The cfDNA concentration in the plasma of mCRPC patients at inclusion in the study correlates with higher blood PSA (without outliers). Abbreviations: Cell-free DNA: cfDNA; Metastatic castration-resistant prostate cancer: mCRPC; Prostate-specific antigen: PSA; Radical prostatectomy: RP.

Figure S5. Determination of whether the cfDNA concentrations in the plasma of different categories of patients correlate with GG at the time of biopsy or at RP. The cfDNA concentrations in the plasma of patients are not associated with GG (outliers were removed). Pathology GG is not available for most mCRPC cases, as several of them did not have an RP. Abbreviations: Cell-free DNA: cfDNA; Gleason grade: GG; Metastatic castration-resistant prostate cancer: mCRPC; Radical prostatectomy: RP.

Figure S6. Determination of whether plasma cfDNA concentrations of RP patients are related to pathological features. The cfDNA concentration in the plasma of patients at the time of RP is not associated with intraductal carcinoma, pT staging, invasion (lymphovascular, lymph nodes, seminal vesicles), and positive margins (outliers were removed). Abbreviations: Cell-free DNA: cfDNA; Metastatic castration-resistant prostate cancer: mCRPC; Not applicable: NA; Pathological lymph node, positive: pN1 and negative: pN0; Pathological tumour: pT; Radical prostatectomy: RP.

Figure S7. Determination of whether plasma cfDNA concentrations of mCRPC patients are related to progression at the time of inclusion. The cfDNA concentration in the plasma of mCRPC patients is not associated with progression at the time of inclusion, irrespective of outliers in the analysis (A) or their removal (B). Although there was a trend for higher median cfDNA concentrations for cases under progression at time of inclusion (P=0.082, without outliers). Abbreviations: Cell-free DNA: cfDNA.

Figure S8. Determination of whether plasma cfDNA concentrations of mCRPC patients are related to progression on treatments. The cfDNA concentration in the plasma of mCRPC patients is not associated with progression on Abiraterone, Docetaxel, and Enzalutamide treatments in the advanced stage of PCa (outliers were removed). Yes = Case had progression on treatment, No = Case did not progress on treatment, NA = Unknown. Abbreviations: Cell-free DNA: cfDNA.

Figure S9. Study of the relationship between plasma cfDNA concentrations and overall survival of mCRPC patients. The cfDNA concentration in the plasma of mCRPC patients is not associated with their overall survival (outliers were removed). Abbreviations: Cell-free DNA: cfDNA.

Figure S10. Chromosomal maps after downsampling high-depth bam files for two cfDNA inputs. The bam files of deep WGS at 147x of 30 ng (A) and 50 ng (B) inputs were downsampled to reach 10x. The procedure was repeated three times, and the results are presented for each of them for the two inputs. Abbreviations: Cell-free DNA: cfDNA; The fraction of the genome that harbors subclonal alterations: Frac.G.subclonal; Tumour fraction: TF.

Legends of Supplementary Tables

Table S1. Literature review of manuscripts referring to cfDNA characterization in prostate cancer. Summary of methods commonly used to isolate and purify cfDNA, prepare libraries for sequencing strategies or other molecular testing, and bioinformatics pipelines. The complete references are indicated.

Table S2. Comparison of replacing AVE buffer for water to elute plasma cfDNA from the Qiagen column. The category of individuals included, elution volumes, concentrations of cfDNA at the first elution, and percentages (%) as well as total are indicated for both AVE and water. The volume of plasma was ~3.6 mL for all samples. Abbreviations: Androgen deprivation therapy: ADT; Cell-free DNA: cfDNA; Metastatic castration-resistant prostate cancer: mCRPC; Radiotherapy: RT.

Table S3. Concentrations of libraries amplified at different PCR cycles. Libraries prepared at the indicated number of cycles for the Standard Reference and PCa cfDNA were aliquoted (triplicates) to determine their concentrations (nM) by qPCR, with the averages also presented. Abbreviations: Cell-free DNA: cfDNA; Quantitative Polymerase Chain Reaction: qPCR.
