# Supplementary material for: Refined Procedure to Purify and Sequence Circulating Cell-Free DNA in Prostate Cancer
Source: Int J Mol Sci. 2025 Jun 18;26(12):5839. doi: 10.3390/ijms26125839 (PMC12192578; doi:10.3390/ijms26125839)
Supplement: Supplementary file 1 [file ijms-26-05839-s001.zip › Supplementary_Table2.pdf]

Sample	Category	First elution volume (µl)	AVE			Water		
			cfDNA per plasma (ng/mL) First elution	cfDNA per plasma (ng/mL) Total	cfDNA recovery Percentage at first elution (%)	cfDNA per plasma (ng/mL) First elution	cfDNA per plasma (ng/mL) Total	cfDNA recovery Percentage at first elution (%)
1	Failed RT case on ADT	60	4.1	4.3	95	7	8.7	80
2	mCRPC	60	2087.8	2119.4	99	318	345.8	92
3	mCRPC	75	33.3	33.3	97	31.7	35.4	90
4	Healthy male	85	10.1	13	77	3.2	3.2	100
5	Healthy male	85	5.6	5.6	100	4.5	4.5	100
6	mCRPC	85	19.3	23.1	84	14.6	15.6	94
7	mCRPC	85	40.3	41.2	98	45.9	48.8	94
