# Supplementary material for: Refined Procedure to Purify and Sequence Circulating Cell-Free DNA in Prostate Cancer
Source: Int J Mol Sci. 2025 Jun 18;26(12):5839. doi: 10.3390/ijms26125839 (PMC12192578; doi:10.3390/ijms26125839)
Supplement: Supplementary file 1 [file ijms-26-05839-s001.zip › Supplementary_Table3.pdf]

Sample	Number of cycles	Concentration by qPCR			Average
		nM	nM	nM	nM ±SD
Standard Reference	8	201.8	219.4	207.0	209.4 ±9.0
	13	202.4	234.3	188.2	208.3 ±23.6
PCa cfDNAs	8	177.2	187.9	185.8	183.6 ±5.7
	10	165.2	185.1	168.3	172.9 ±10.7
	13	238.8	239.8	236.6	238.4 ±1.7
