# Supplementary figures and images for: Refined Procedure to Purify and Sequence Circulating Cell-Free DNA in Prostate Cancer
Source: Int J Mol Sci. 2025 Jun 18;26(12):5839. doi: 10.3390/ijms26125839 (PMC12192578; doi:10.3390/ijms26125839)

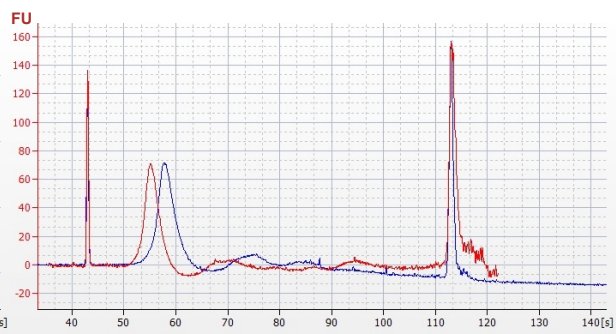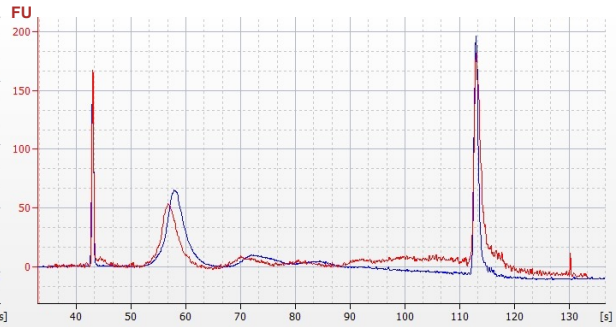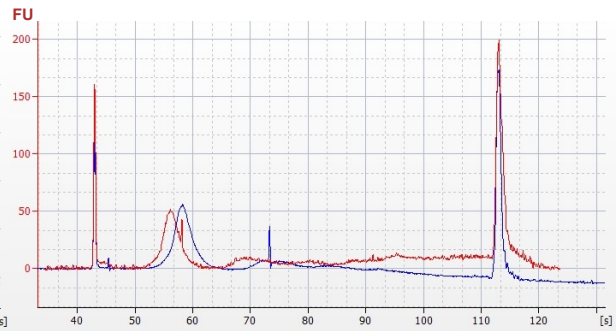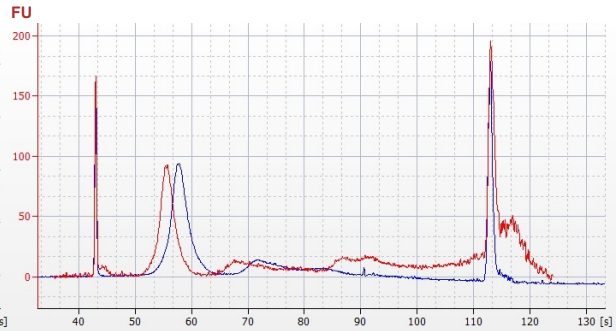

Supplement: Supplementary file 1 [file ijms-26-05839-s001.zip › Supplementary_Figure1.pdf]

# A 30 ng input cfDNA – Downsampled 10x

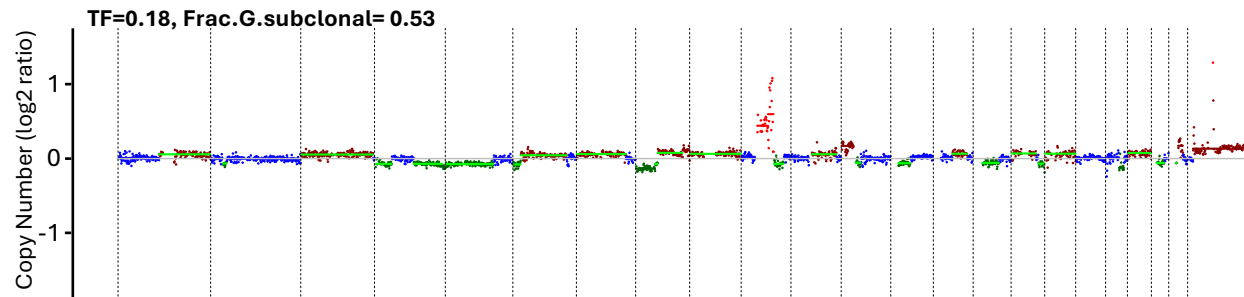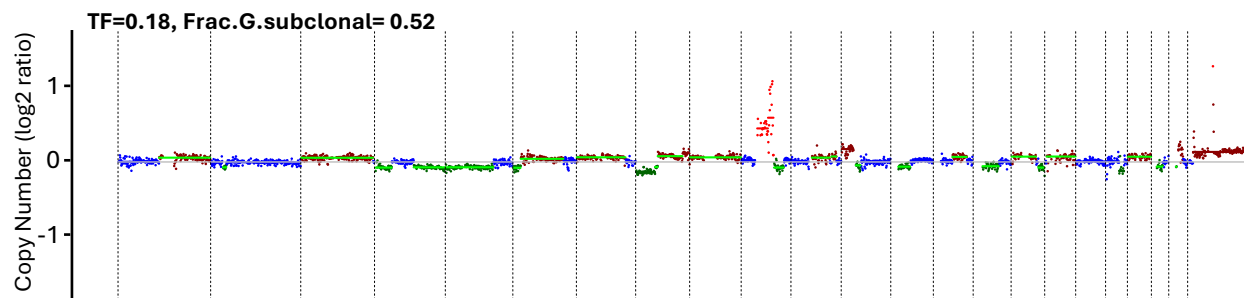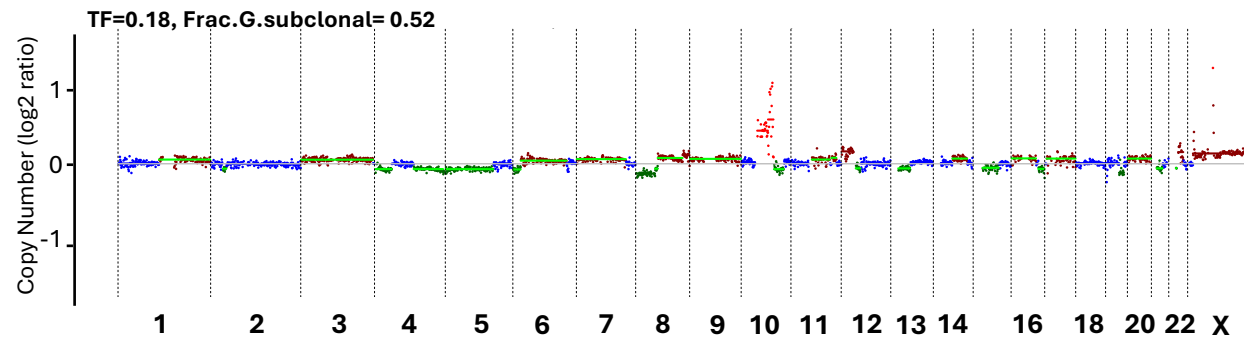

# B 50 ng input cfDNA – Downsampled 10x

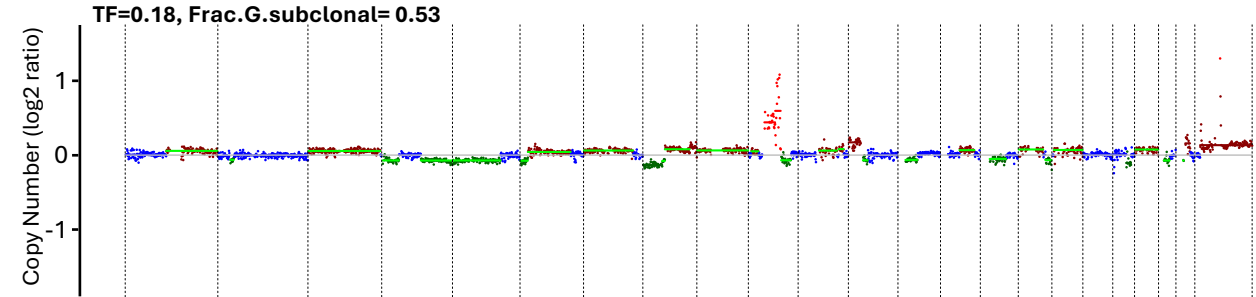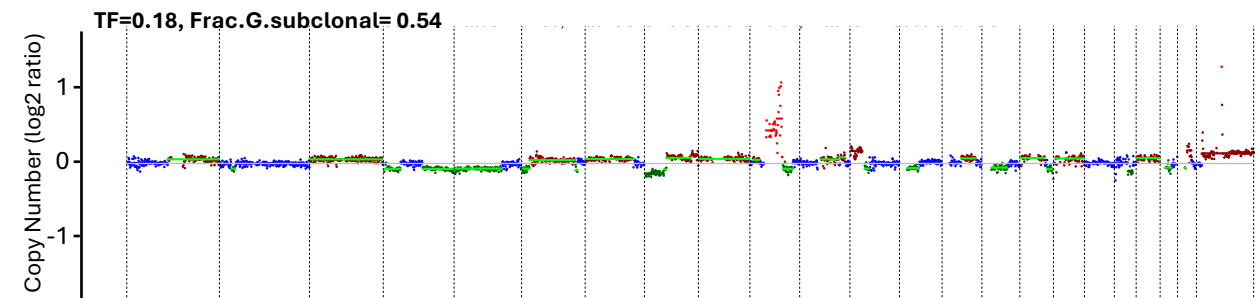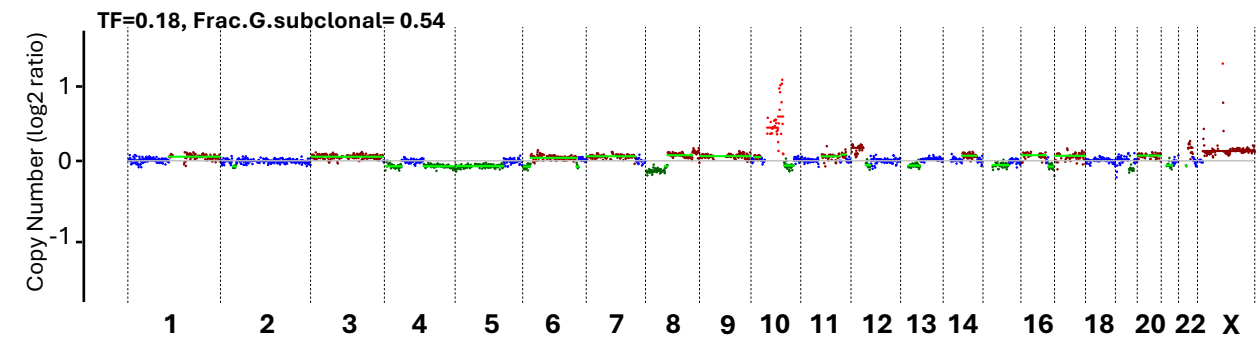

Supplement: Supplementary file 1 [file ijms-26-05839-s001.zip › Supplementary_Figure10.pdf]

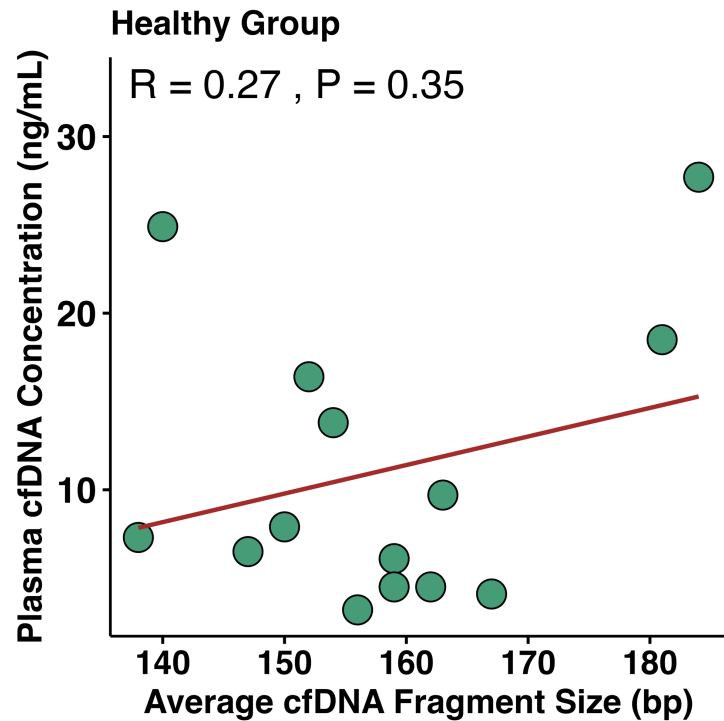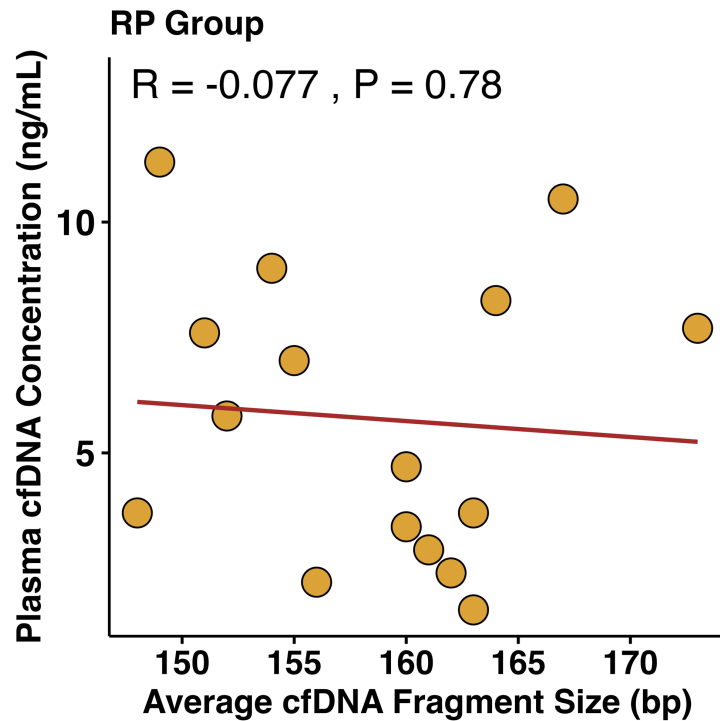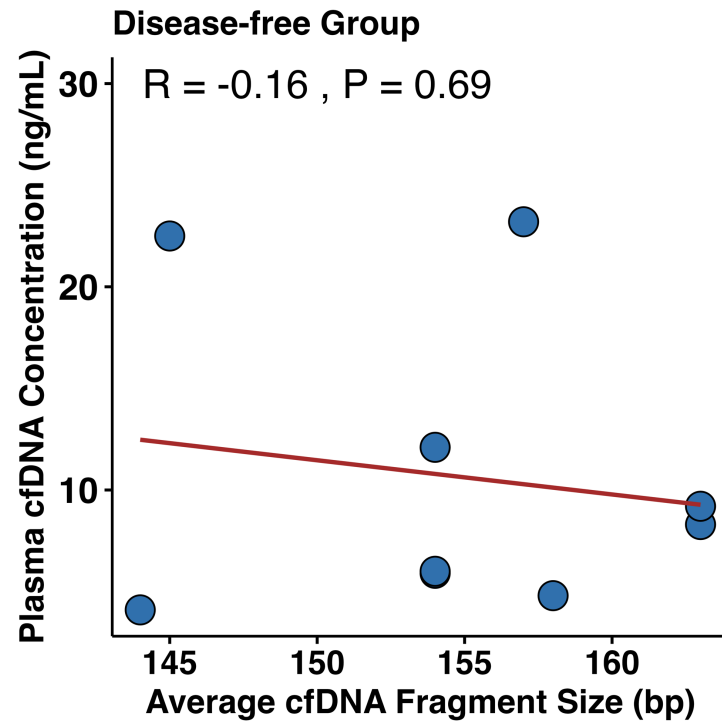

Supplement: Supplementary file 1 [file ijms-26-05839-s001.zip › Supplementary_Figure2.pdf]

**A**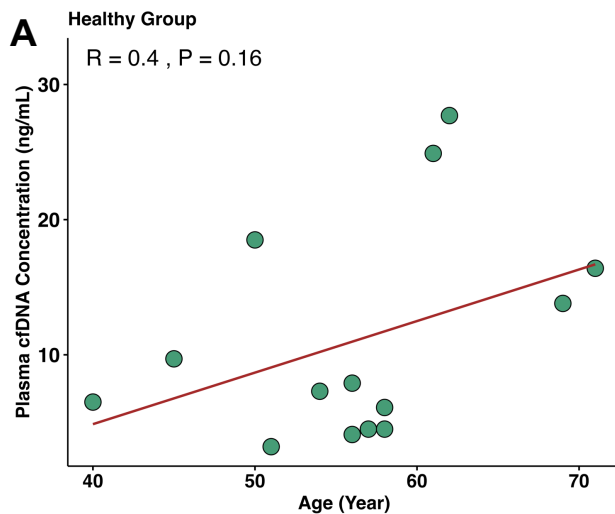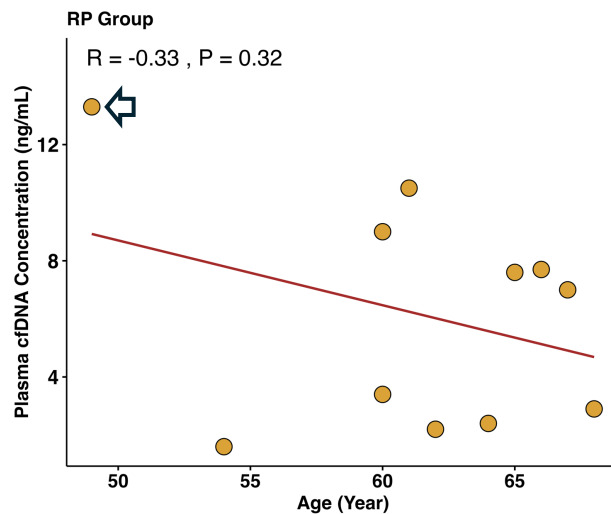**B**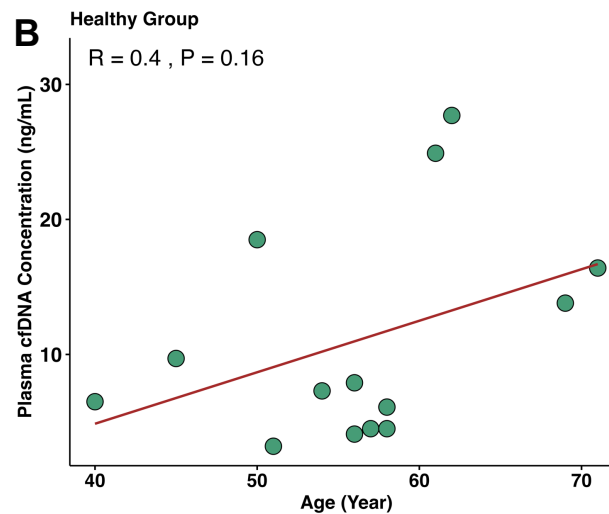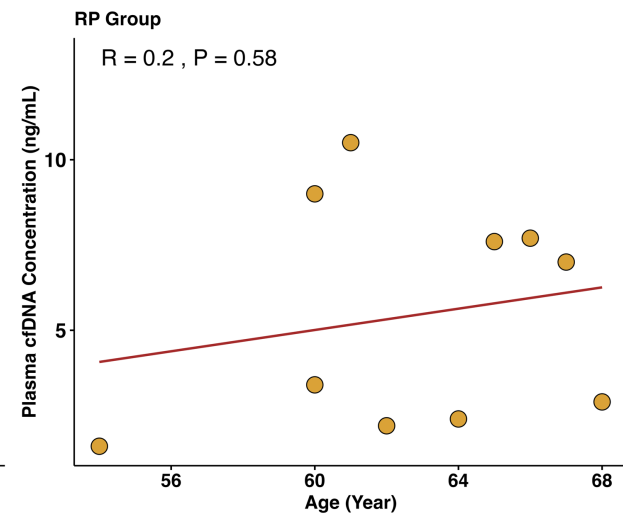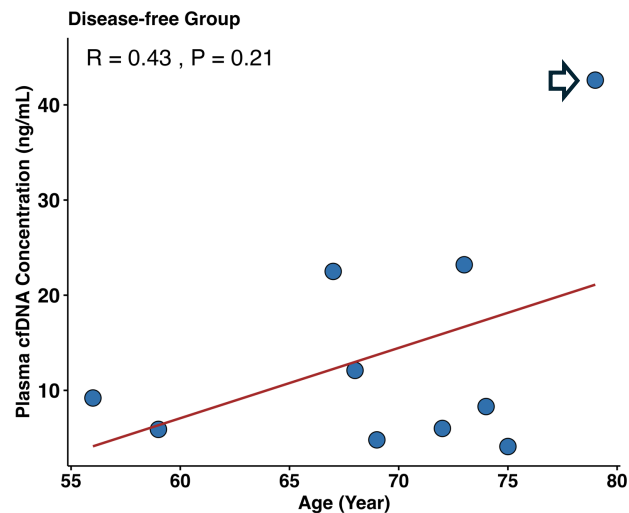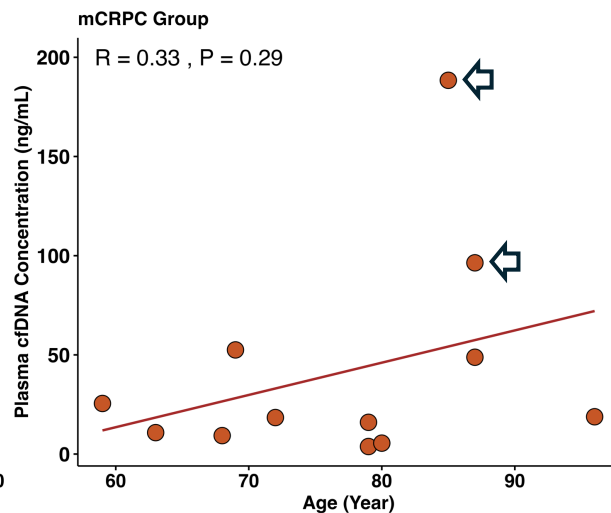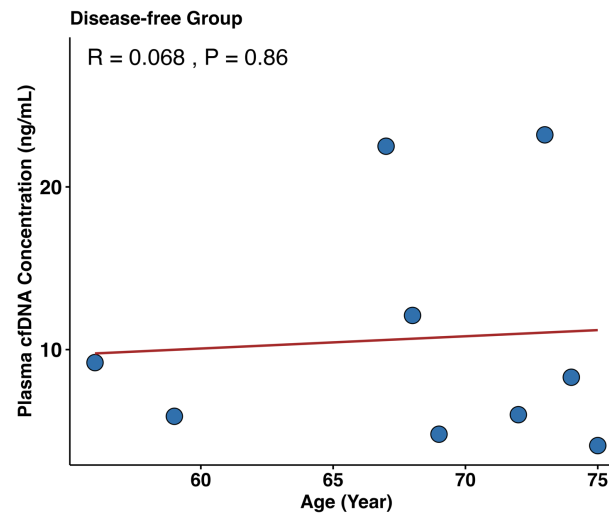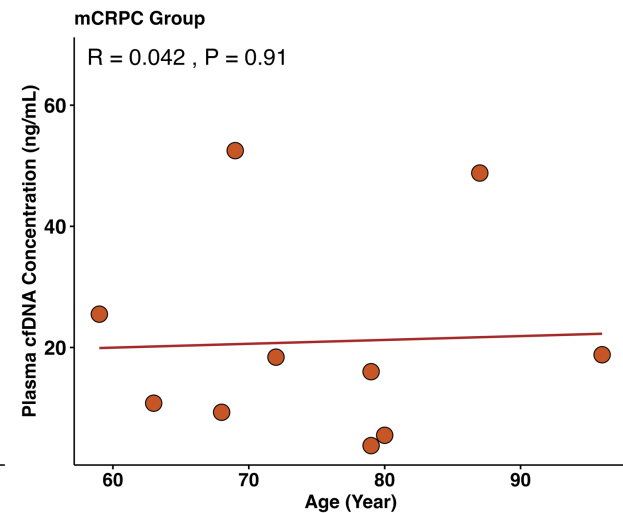

Supplement: Supplementary file 1 [file ijms-26-05839-s001.zip › Supplementary_Figure3.pdf]

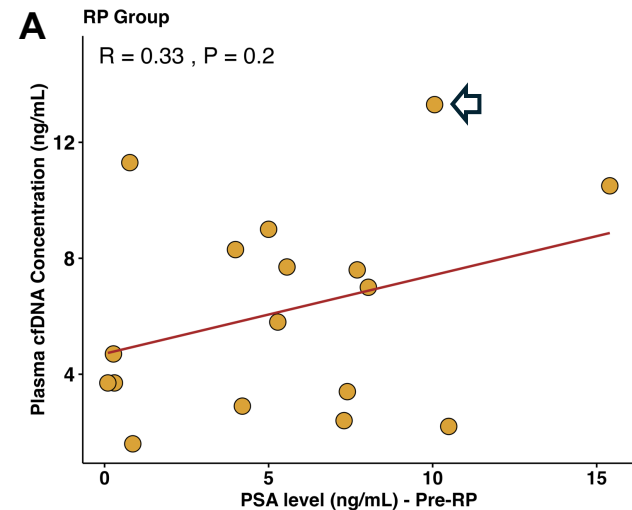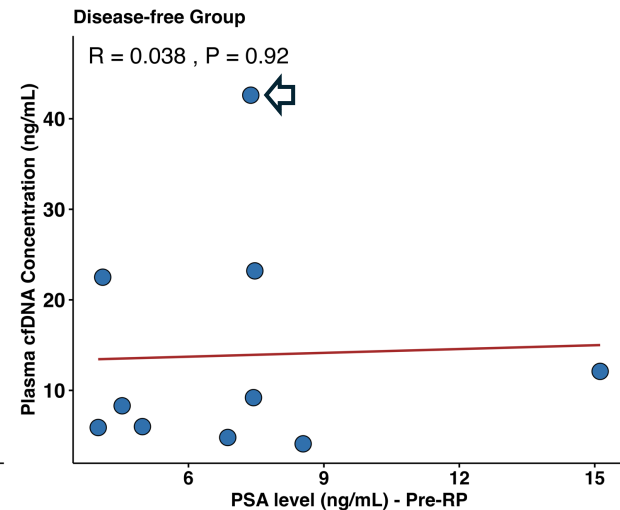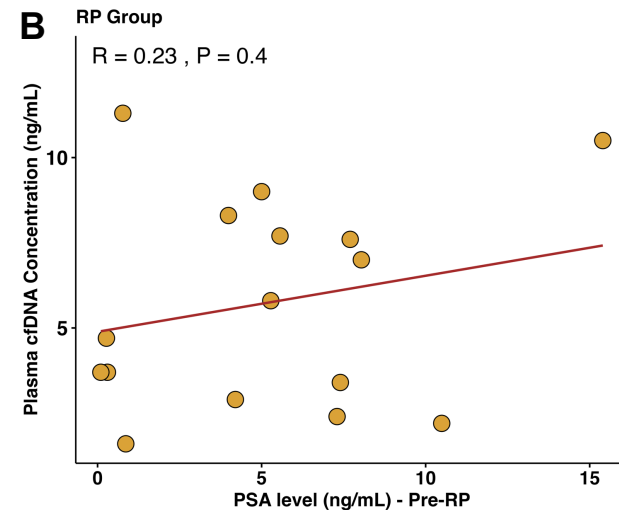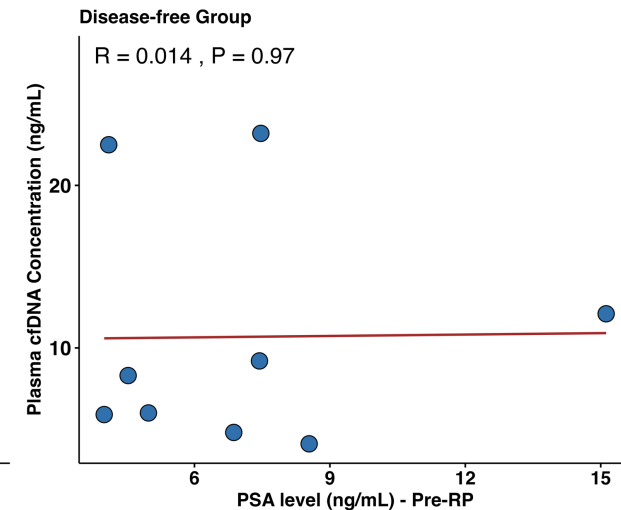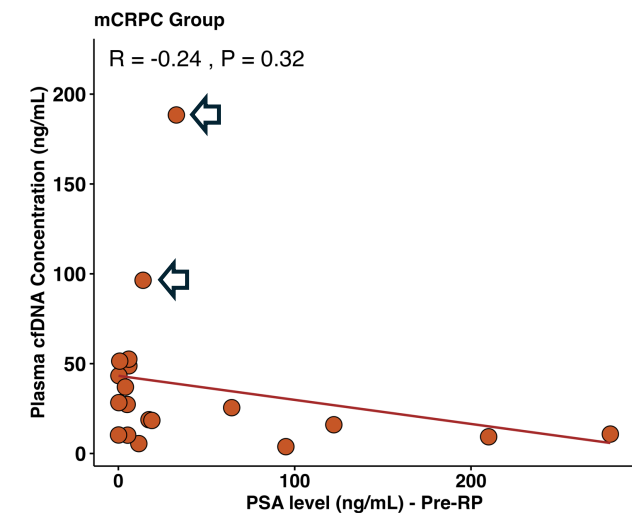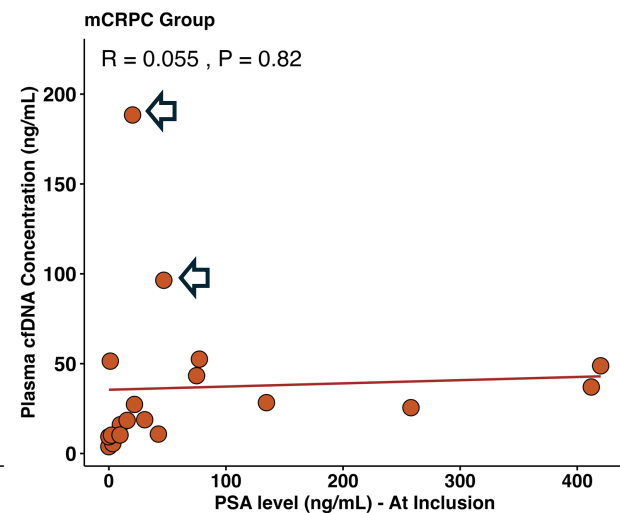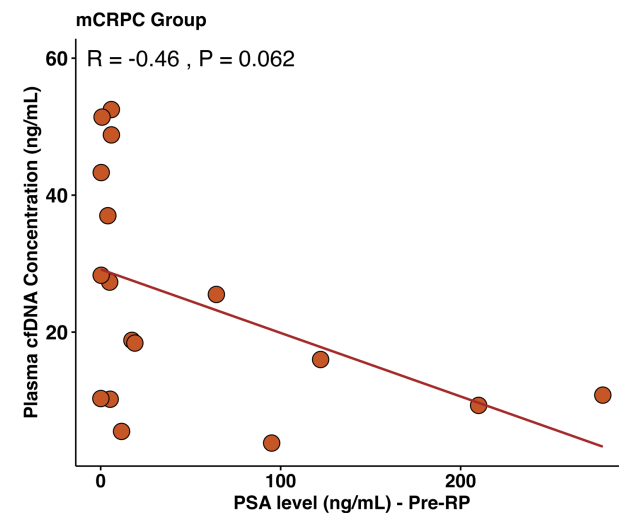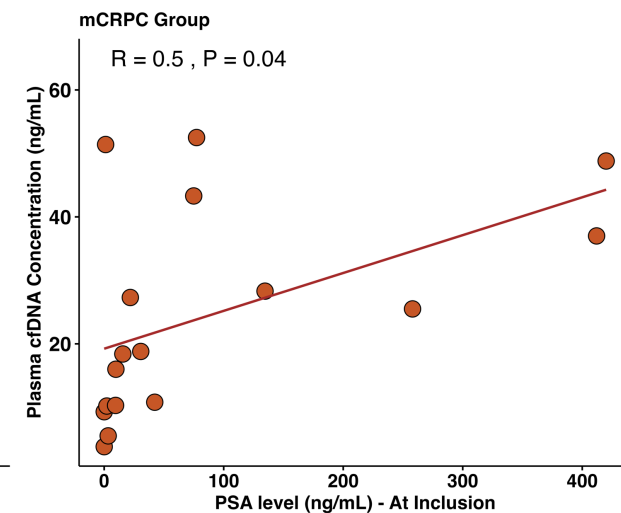

Supplement: Supplementary file 1 [file ijms-26-05839-s001.zip › Supplementary_Figure4.pdf]

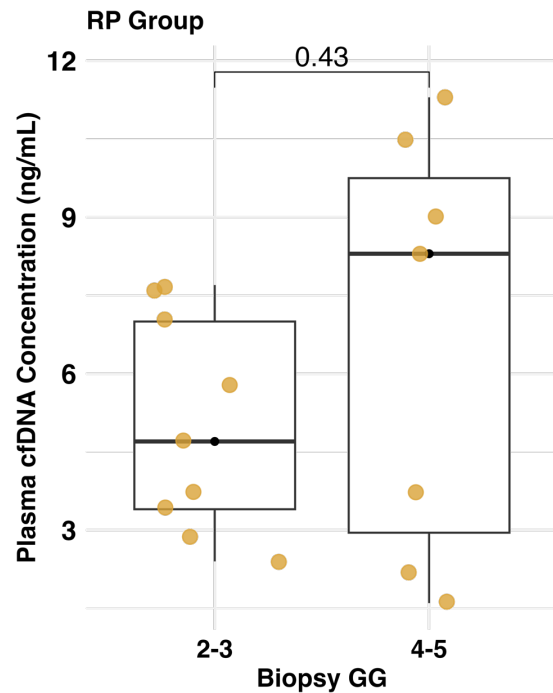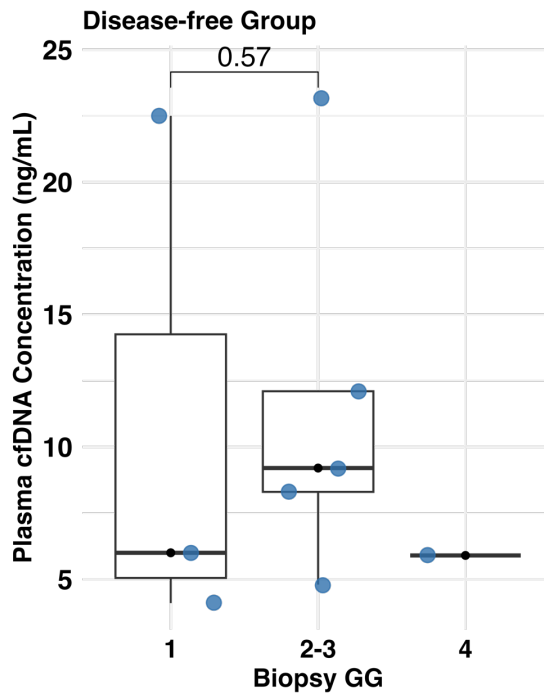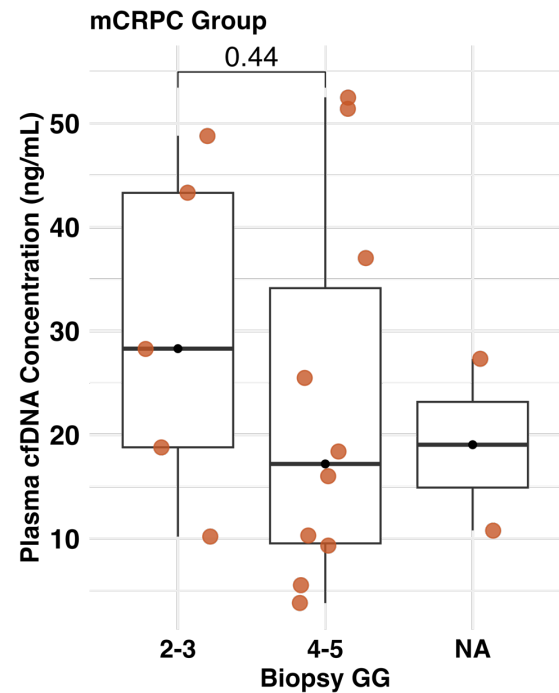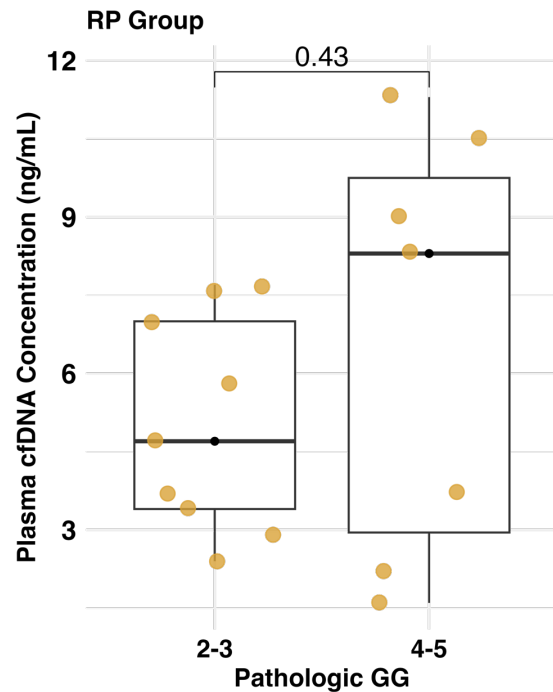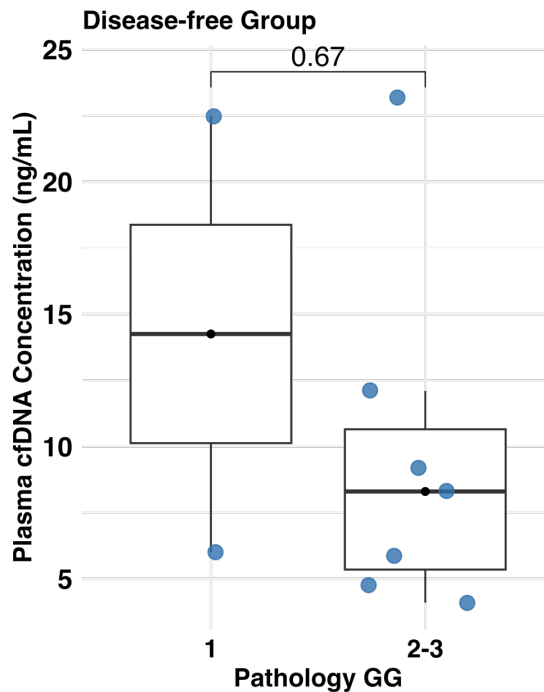

Supplement: Supplementary file 1 [file ijms-26-05839-s001.zip › Supplementary_Figure5.pdf]

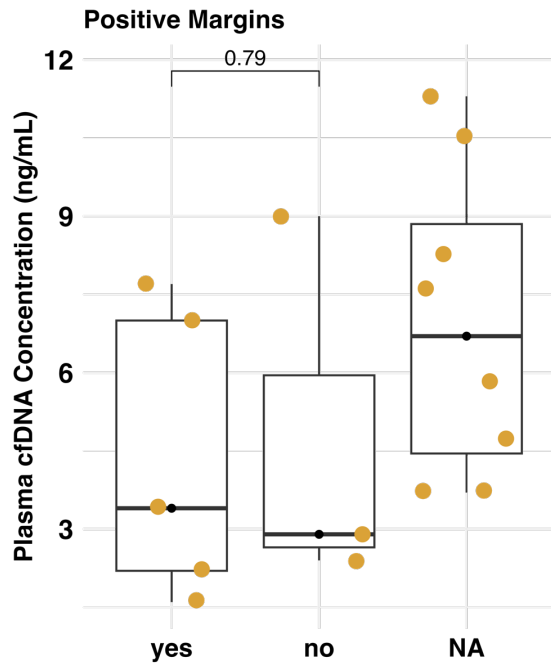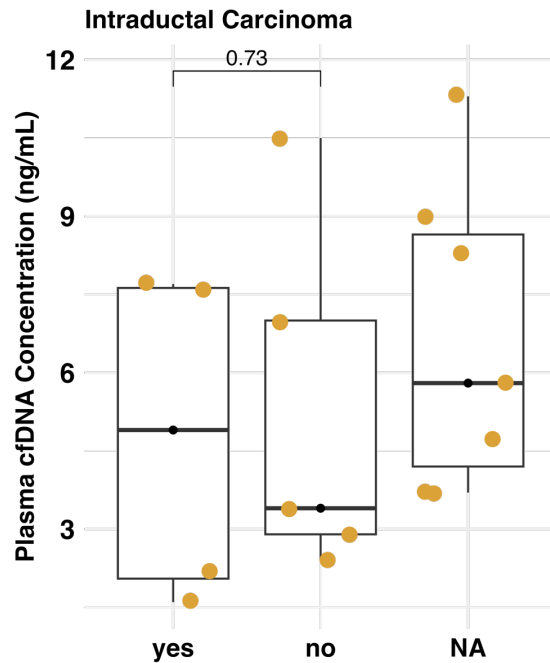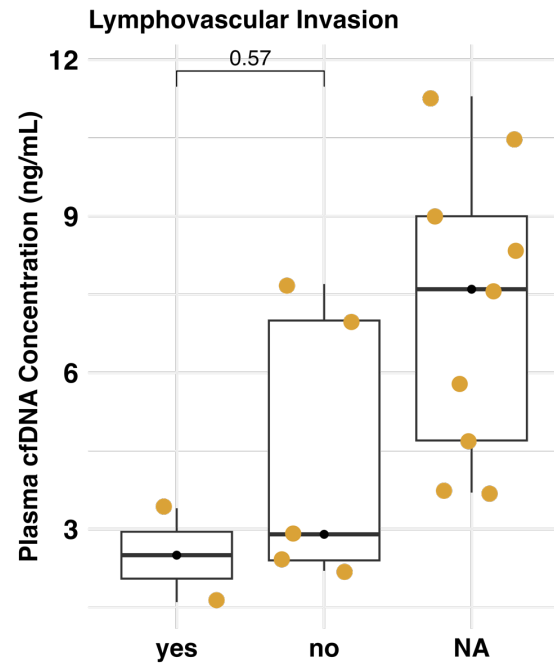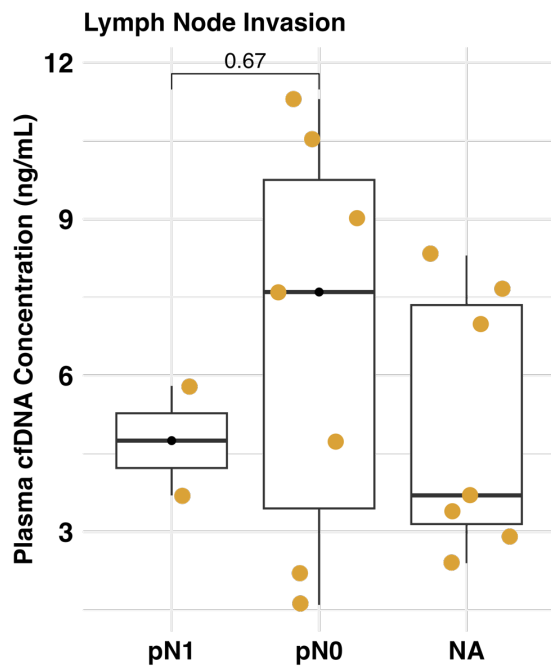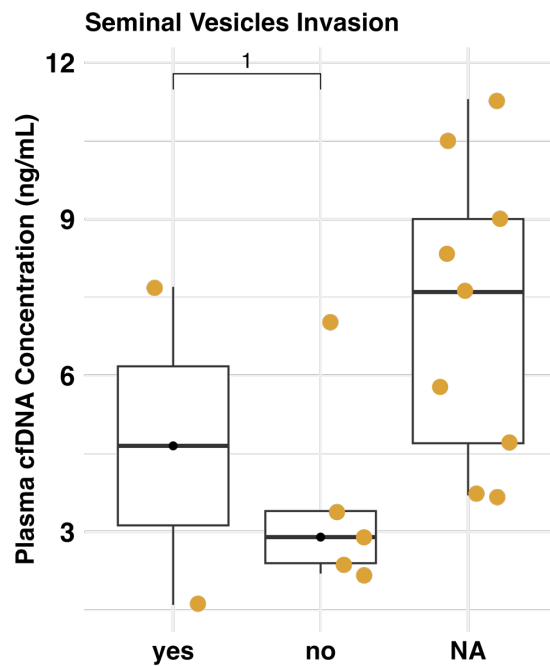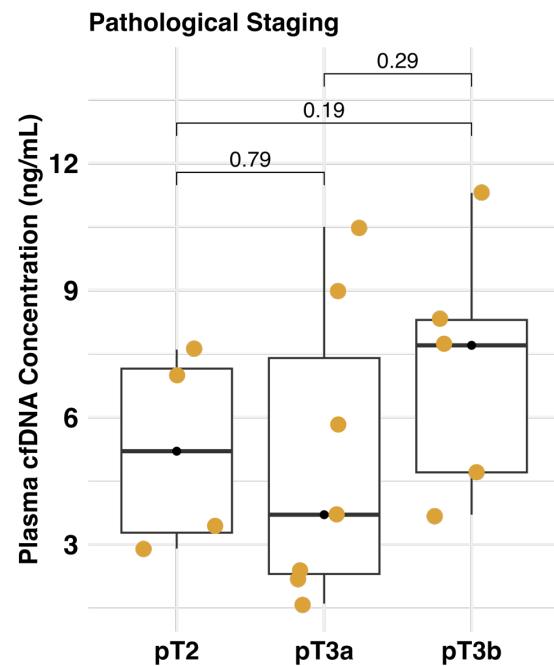

Supplement: Supplementary file 1 [file ijms-26-05839-s001.zip › Supplementary_Figure6.pdf]

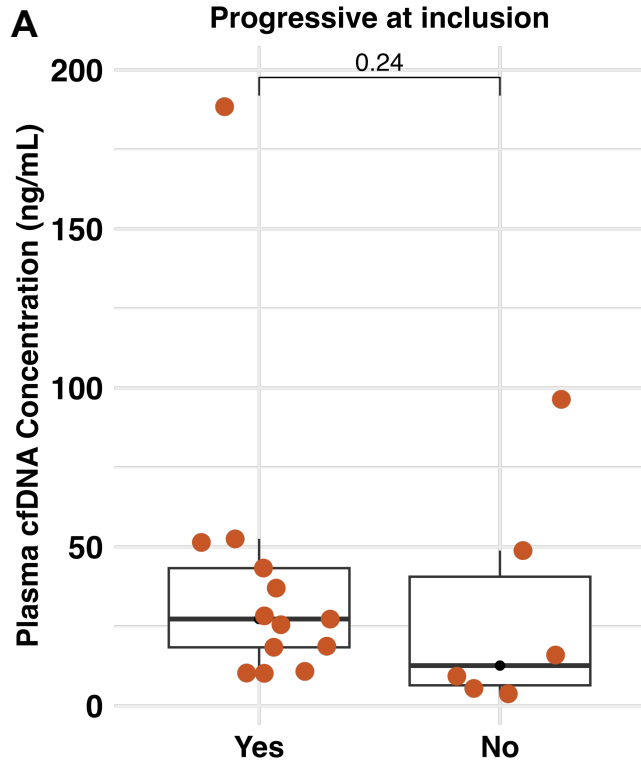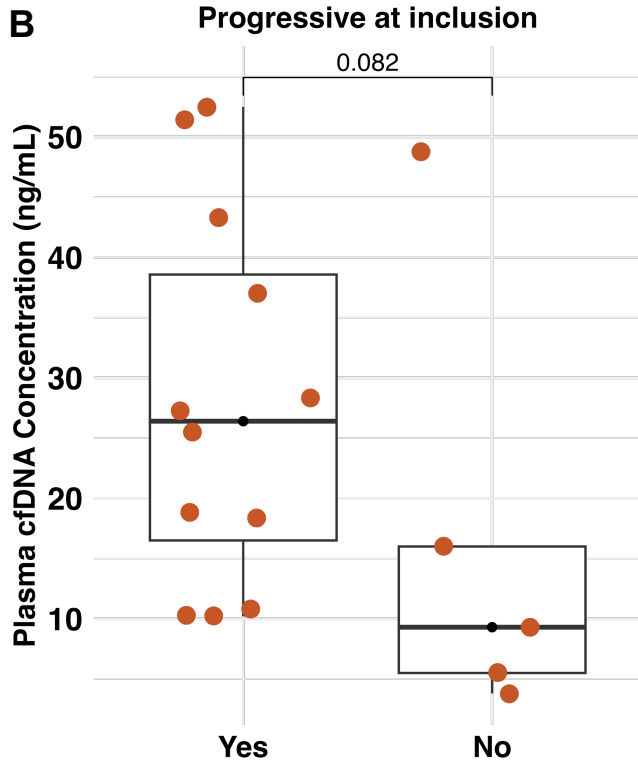

Supplement: Supplementary file 1 [file ijms-26-05839-s001.zip › Supplementary_Figure7.pdf]

Progression on Abiraterone

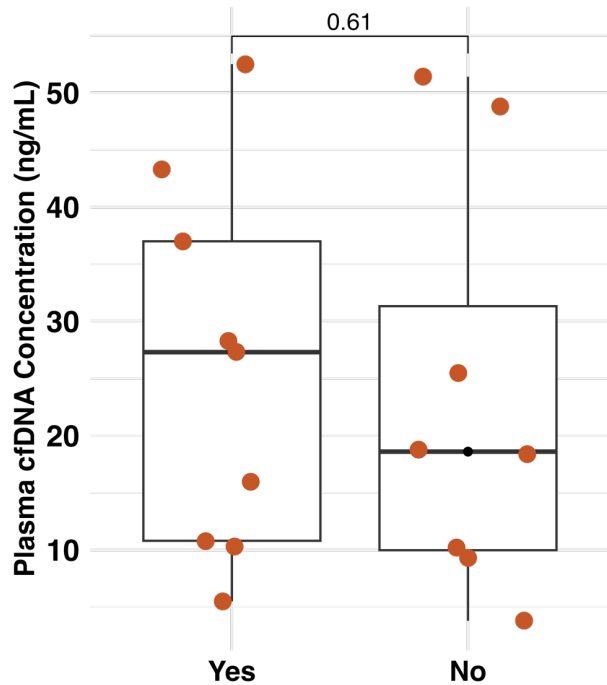

Progression on Docetaxel

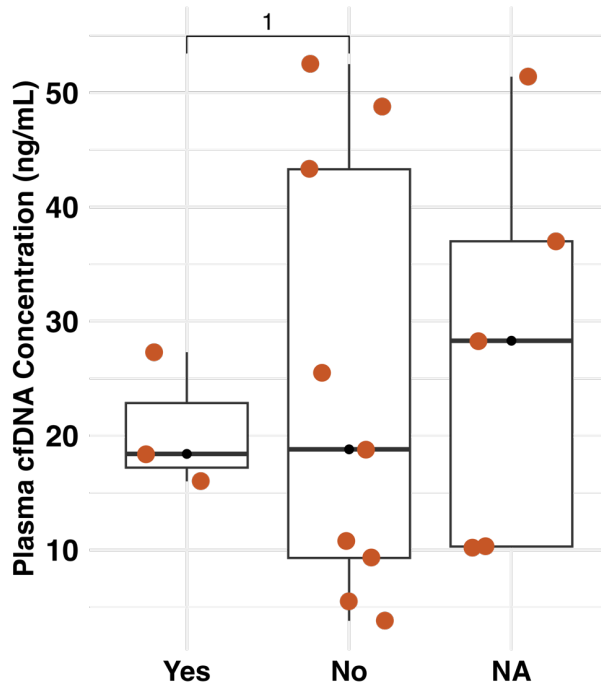

Progression on Enzalutamide

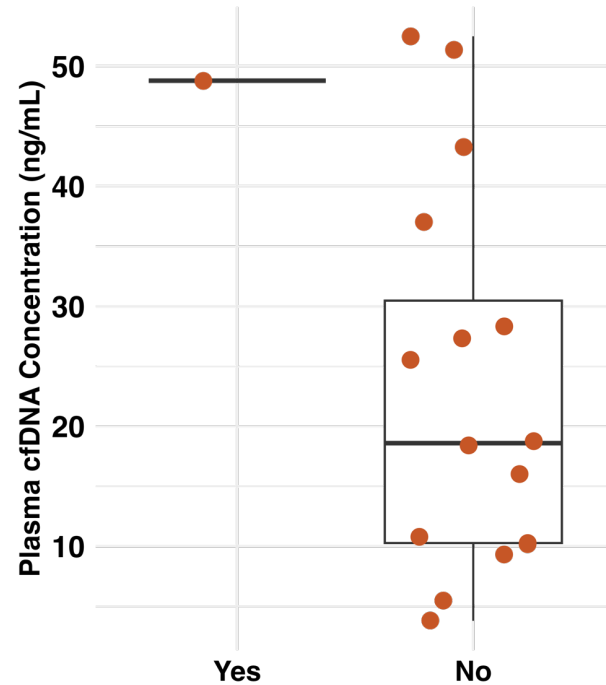

Supplement: Supplementary file 1 [file ijms-26-05839-s001.zip › Supplementary_Figure8.pdf]

## Overall Survival

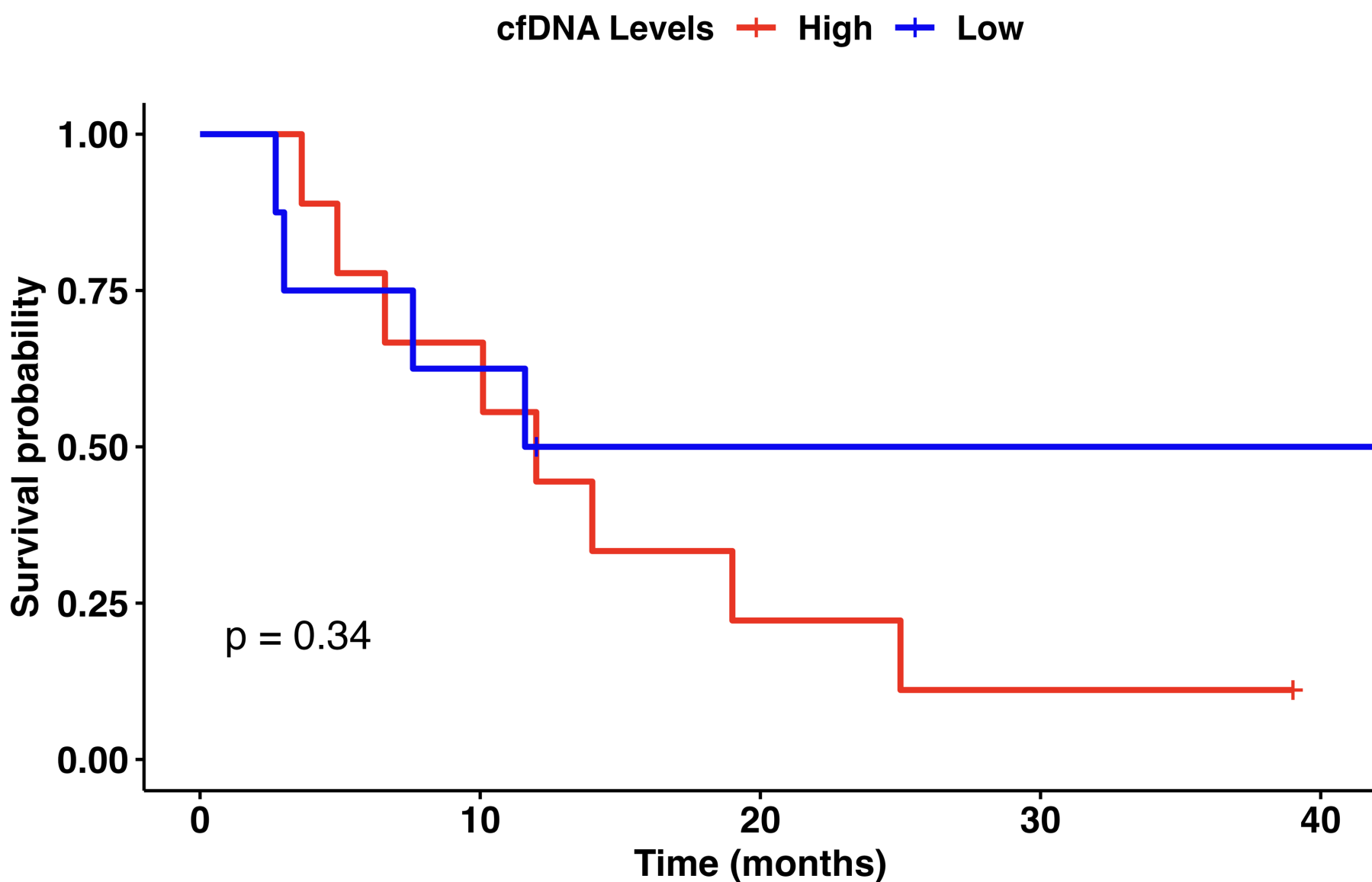

## Number at risk of death

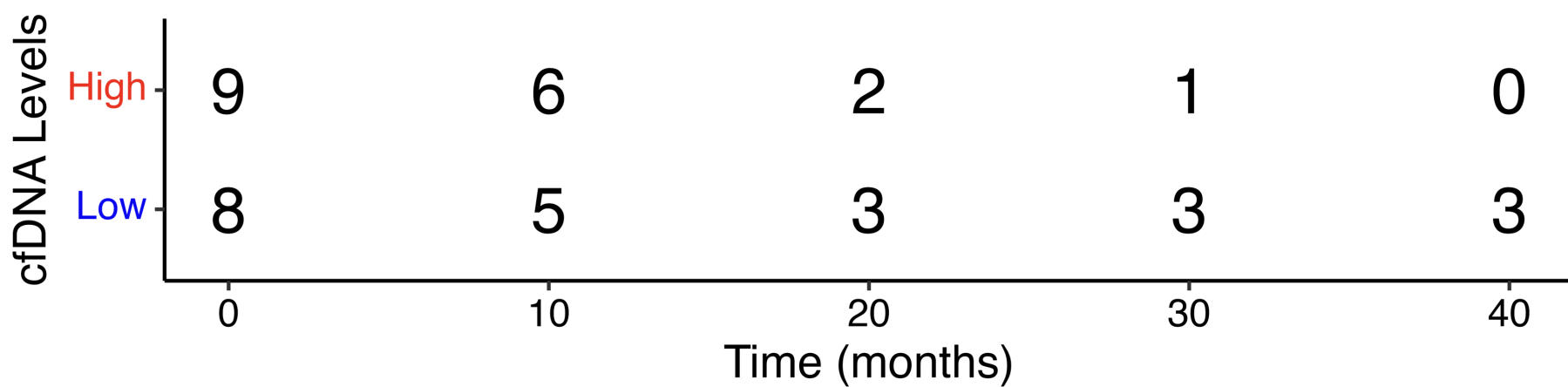

Supplement: Supplementary file 1 [file ijms-26-05839-s001.zip › Supplementary_Figure9.pdf]
